# The British Society of Gastroenterology/UK-PBC primary biliary cholangitis treatment and management guidelines

**DOI:** 10.1136/gutjnl-2017-315259

**Published:** 2018-03-28

**Authors:** Gideon M Hirschfield, Jessica K Dyson, Graeme J M Alexander, Michael H Chapman, Jane Collier, Stefan Hübscher, Imran Patanwala, Stephen P Pereira, Collette Thain, Douglas Thorburn, Dina Tiniakos, Martine Walmsley, George Webster, David E J Jones

**Affiliations:** 1 NIHR Birmingham Biomedical Research Centre, Birmingham, UK; 2 University Hospitals Birmingham NHS Foundation Trust, Birmingham, UK; 3 Centre for Liver Research, Institute of Immunology and Immunotherapy, University of Birmingham, Birmingham, UK; 4 Newcastle upon Tyne Hospitals NHS Foundation Trust, Newcastle upon Tyne, UK; 5 Institute of Cellular Medicine, Newcastle University, Newcastle upon Tyne, UK; 6 NIHR Newcastle Biomedical Research Centre, Newcastle, United Kingdom; 7 Sheila Sherlock Liver Centre, Royal Free London NHS Foundation Trust, London, UK; 8 UCL Institute for Liver and Digestive Health, Division of Medicine, University College London, London, UK; 9 Department of Gastroenterology, University College London Hospitals NHS Foundation Trust, London, UK; 10 Translational Gastroenterology Unit, Oxford University Hospitals, University of Oxford, Oxford, UK; 11 Department of Cellular Pathology, University Hospitals Birmingham NHS Foundation Trust, Birmingham, UK; 12 Department of Gastroenterology, Royal Liverpool and Broadgreen University Hospitals NHS Trust, Liverpool, UK; 13 University of Liverpool, Liverpool, UK; 14 PBC Foundation, Edinburgh, UK; 15 PSC Support, Didcot, UK

**Keywords:** autoimmune liver disease, care pathway, guidelines, ursodeoxycholic acid, obeticholic acid

## Abstract

Primary biliary cholangitis (formerly known as primary biliary cirrhosis, PBC) is an autoimmune liver disease in which a cycle of immune mediated biliary epithelial cell injury, cholestasis and progressive fibrosis can culminate over time in an end-stage biliary cirrhosis. Both genetic and environmental influences are presumed relevant to disease initiation. PBC is most prevalent in women and those over the age of 50, but a spectrum of disease is recognised in adult patients globally; male sex, younger age at onset (<45) and advanced disease at presentation are baseline predictors of poorer outcome. As the disease is increasingly diagnosed through the combination of cholestatic serum liver tests and the presence of antimitochondrial antibodies, most presenting patients are not cirrhotic and the term cholangitis is more accurate. Disease course is frequently accompanied by symptoms that can be burdensome for patients, and management of patients with PBC must address, in a life-long manner, both disease progression and symptom burden. Licensed therapies include ursodeoxycholic acid (UDCA) and obeticholic acid (OCA), alongside experimental new and re-purposed agents. Disease management focuses on initiation of UDCA for all patients and risk stratification based on baseline and on-treatment factors, including in particular the response to treatment. Those intolerant of treatment with UDCA or those with high-risk disease as evidenced by UDCA treatment failure (frequently reflected in trial and clinical practice as an alkaline phosphatase >1.67 × upper limit of normal and/or elevated bilirubin) should be considered for second-line therapy, of which OCA is the only currently licensed National Institute for Health and Care Excellence recommended agent. Follow-up of patients is life-long and must address treatment of the disease and management of associated symptoms.

## Executive summary

Primary biliary cholangitis (PBC) is a chronic autoimmune liver disease. It continues to have a burden of morbidity and mortality that spans both the consequences of a sometimes progressive biliary injury, alongside a symptom profile notably encompassing pruritus, sicca complex, fatigue, abdominal discomfort and arthralgias/bone pain. UK-PBC and the British Society of Gastroenterology (BSG) have partnered to develop a comprehensive guideline document to provide detailed advice and recommendations on the best approaches to the management of the disease. A series of recommendations and audit standards are proposed to ensure that patients are offered timely licensed therapy (ursodeoxycholic acid (UDCA), obeticholic acid (OCA)) in addition to being actively managed for symptoms as well as complications of progressive liver disease.

In brief its key recommendations, based on the GRADE classification system (Strong/Weak; quality of evidence: High/Moderate/Low/Very low), are:

1. The presence of antimitochondrial antibodies (>1 in 40) or highly PBC-specific antinuclear antibodies, in the appropriate context of cholestatic liver biochemistry, without alternative explanation, is usually sufficient for confidently reaching the diagnosis of PBC (Strong; High).
2. All patients with PBC should be offered structured life-long follow-up, recognising that different patients have different disease courses and may require different intensity of follow-up (Strong; Moderate).
3. Risk assessment should evaluate disease severity and activity at baseline and on treatment. We recommend a combination of serum liver tests (to identify those with an elevated bilirubin, a platelet count <150 or biochemical disease activity on treatment), imaging (liver ultrasound to identify overt cirrhosis and splenomegaly; transient elastography to identify increased liver stiffness) and recognition of young age at disease onset (<45 years) and male sex (Strong; Moderate).
4. To identify those at greatest risk of disease progression, we recommend that all patients have individualised risk stratification using biochemical response indices following 1 year of UDCA therapy (Strong; High). We suggest that UDCA treated patients with an alkaline phosphatase (ALP) >1.67 x upper limit of normal (ULN) and/or elevated bilirubin <2 x ULN represent a group of high-risk patients in whom there is randomised controlled trial evidence for the addition of second-line therapy (Weak; Moderate).
5. We recommend oral UDCA at 13–15 mg/kg/day is used as the first-line pharmacotherapy in all patients with PBC. If tolerated, treatment should usually be life-long (Strong; High).
6. In patients with inadequate response to UDCA (or UDCA intolerance) as defined by ALP >1.67 x ULN and/or elevated bilirubin <2 x ULN, the addition of OCA has been associated with improvements in biochemical surrogates of disease activity reasonably likely to predict improved outcomes. We therefore recommend, in keeping with the NICE evaluation of OCA, that the addition of OCA for patients with an inadequate response to UDCA, or intolerant of UDCA, is considered. We recommend dose adjustment in patients with advanced liver disease as per the drug label (Strong; Low).
7. We recommend all patients should be evaluated for the presence of symptoms, particularly fatigue and itch. Clinicians should recognise that severity of symptoms does not correlate with stage of disease (Strong; Moderate).
8. True overlap with autoimmune hepatitis is probably rare and we suggest that, when suspected, liver biopsy with expert clinicopathological review is needed to make the diagnosis and guide treatment (Strong; Moderate).
9. We recommend that patients with PBC should be offered the chance to seek support from patient support groups (Strong; Moderate).
10. We recommend that clinicians caring for patients with PBC should consider introducing clinical audit tools to document and improve the quality of care delivered to patients (Strong; Low).

## Introduction

Primary biliary cholangitis (formerly known as primary biliary cirrhosis, PBC), is a life-long autoimmune cholestatic liver disease that is a rare but important cause of chronic liver disease. More than 15 000 individuals in the UK live with the risks and consequences of chronic biliary inflammation. New advances in clinical disease understanding have highlighted individual risk, and demonstrated the value to patients of approaches to risk stratification. At present, care remains predominantly led by secondary and tertiary care physicians, who confirm diagnosis, initiate therapy and coordinate ongoing follow-up. These guidelines are targeted predominantly towards those gastroenterologists and hepatologists leading the care of patients with PBC. However, in addition they will be of value to nurses, primary care physicians and those more broadly involved in patient care, as well as patients themselves. The guidelines have been developed as a partnership between the BSG and UK-PBC, a Medical Research Council (MRC)-funded National Institute for Health Research (NIHR) Rare Disease adopted, stratified medicine initiative in PBC (www.uk-pbc.com). The guideline development has followed the BSG established pathway (http://www.bsg.org.uk/images/stories/docs/clinical/guidelines/general/bsg_guidelines_advice_document_may2016.pdf),^1^ and includes development of a broad membered cholestasis Guidelines Development Group, including patient participation.

The impact for patients living with PBC reflects the risk of development of advanced cirrhotic and portal hypertensive liver disease as well as marked effects on quality of life (QoL) from associated symptoms. Treatment is available for patients with PBC and some of its symptoms, increasing the importance of timely evaluation and diagnosis. Stratification of personal risk of complications is emerging and highlights the ‘at-risk’ individuals for whom additional new therapies may ultimately be suitable.

Diagnostically, PBC should always be considered in patients with otherwise unexplained repeated elevation of usually serum alkaline phosphatase (ALP), but also gamma-glutamyl transferase (GGT). Autoantibody status should be checked in all such patients and the presence of clinically significant antimitochondrial antibody (AMA or anti-M2 ELISA according to local practice) is sufficient to confirm the diagnosis in the absence of biopsy in most patients. The presence of specific anti-nuclear-rim, anti-nuclear-dot or anti-centromere antibodies (or anti-gp210 or sp-100 by ELISA) can frequently be sufficient to diagnose AMA-negative PBC. True autoantibody-negative disease exists and can only be diagnosed on biopsy.

Oral ursodeoxycholic acid (UDCA) therapy is appropriate for all patients at a dose of 13–15 mg/kg/day. Crossover features suggestive of a potentially corticosteroid-responsive autoimmune hepatitis-type liver injury should be considered in patients only after further investigation, usually including a liver biopsy and expert hepatopathological review. Inadequate response to UDCA (defined using validated criteria) has been robustly associated with increased risk of death or need for liver transplantation. The concept of treatment failure with UDCA is evolving and no single risk tool has been identified as ideal; however, the concept that the lower the serum ALP value, the better the patient outcome is reflected in all tools, alongside other predictive factors such as bilirubin, age and platelet count. Those classified by their clinicians as having an inadequate response to UDCA have a clear enhanced risk of liver disease progression, and in particular such patients should be subject to long-term monitoring for the complications of cirrhosis. At the time of writing, although there are numerous risk scores proposed for patients with PBC, there is insufficient evidence to recommend one over another on the grounds of head-to-head data; each stratifier as discussed has, however, been validated. Despite this, it should be noted that the ‘Toronto’ biochemical stratification (an ALP value of at least 1.67 times the upper limit of the normal (ULN) range and/or an abnormal total bilirubin) has been used in clinical trial settings and represents a simple and easily applied stratifier of risk for clinicians and patients. Second-line therapy in the UK has been licensed and recommended by the National Institute for Health and Care Excellence (NICE) in the form of obeticholic acid (OCA). Patients failing UDCA, or those intolerant of UDCA, therefore now have the opportunity to consider (conditionally) licensed therapy other than UDCA. In addition, other therapies (repurposed and new) continue to also be evaluated.

Given the heightened awareness of poorer outcomes, attention should be given to managing high-risk, younger and UDCA non-responsive patients in specialist centres. Deterioration of PBC can be rapid in the end stages (particularly once a patient is jaundiced) and timely referral for consideration of transplantation, which is an effective treatment for end-stage disease, is essential. Recurrence of disease post-transplant is reported, but only rarely clinically relevant.

While the majority of patients will have good QoL, for a significant and important minority, impairment is notable and clinicians should enquire specifically about symptoms. Cholestatic pruritus affects about a third of patients and effective first-line (bile acid sequestrants) and second-line (rifampicin) therapies exist, although with tolerability and side effect concerns. Fatigue is a significant problem in up to half of patients and is complex in nature. Social isolation is an important factor in poor QoL in fatigued patients with PBC. There is no single effective therapy for fatigue and a structured approach, including effective treatment of comorbid conditions such as pruritus (nocturnal itch can be a significant factor in sleep disturbance contributing to fatigue) and depression, is needed.

## Guideline development process

These guidelines are designed primarily with the hospital physician in mind. They nevertheless underpin the management of PBC across all specialities and between primary and hospital care. The guidelines have been produced as a consensus document of the BSG Liver Section and UK-PBC with the aim of assisting clinicians in the diagnosis and management of patients with PBC. The guidelines were initiated by the Liver Section of the BSG and approved by the BSG Clinical Services and Standards Committee (CSSC), with internal peer review by the BSG. Members of the writing committee included gastroenterologists, hepatologists, transplant physicians, liver pathologists and patient representatives. Additional review has been sought from experts spanning primary and secondary care, as well as patient charities. Where possible, clear, clinically applicable recommendations are provided.

### Guidelines Development Group (GDG)

The Guidelines Development Group (which met twice in person and regularly by email) had a broad constitution. All members declared their conflicts of interest to the BSG prior to guideline writing. Consensus was reached for therapeutic guidance where perceived conflicts were possible. Feedback was received from the British Liver Trust, LIVErNORTH, Royal College of General Practitioners, Nurse Representation (Sam Ducker) and the British Association for the Study of the Liver, as well as the Liver Section of the BSG. In addition to this, draft guidelines were posted on the UK-PBC website for a time limited period for open comment.

These guidelines have been produced using a systematic review of publications identified using PubMed, Medline and Cochrane database searches in line with the Appraisal of Guidelines Research & Evaluation (AGREE) instrument II (www.agreetrust.org). The primary keywords for baseline searches (completed in June 2017) were ‘primary biliary cirrhosis’, ‘primary biliary cholangitis’ and ’autoimmune overlap syndrome’. Additional keywords were included for specific searches such as ‘therapy’ and ‘ursodeoxycholic acid’.

### Evidence levels (as per Grading of Recommendations, Assessment, Development and Evaluations (GRADE) system)

The recommendations are based on the GRADE classification system (Strong/Weak; quality of evidence: High/Moderate/Low/Very low).

GRADE classifies recommendations as strong or weak. Strength of recommendation is determined by the balance between desirable and undesirable consequences of alternative management strategies, quality of evidence, variability in values and preferences, and resource use. The larger the difference between the desirable and undesirable effects, the higher the likelihood that a strong recommendation is warranted. The narrower the gradient, the higher the likelihood that a weak recommendation is warranted. The higher the quality of evidence, the higher the likelihood that a strong recommendation is warranted. The more values and preferences vary, or the greater the uncertainty in values and preferences, the higher the likelihood that a weak recommendation is warranted. The higher the costs of an intervention—that is, the greater the resources consumed—the lower the likelihood that a strong recommendation is warranted. Strong recommendations mean that most informed patients would choose the recommended management and that clinicians can structure their interactions with patients accordingly. Weak recommendations mean that patients’ choices will vary according to their values and preferences, and clinicians must ensure that patients’ care is in keeping with their values and preferences.

## Background

PBC is a chronic autoimmune cholestatic liver disease.^2 3^ Previous guidelines have included the European Association for the Study of the Liver (EASL) and American Association for the Study of Liver Diseases (AASLD) practice guidelines which review prior literature and cite many important references.^4–6^ These current guidelines build from previous documents and include an approach to the management of PBC wherein care is delivered to patients based on individual risk of disease-associated complications.

The characteristics of PBC are sustained elevation (>6 months) above the ULN for serum ALP activity, the presence of frequently granulomatous inflammation of the portal tracts accompanying lymphocytic mediated damage to (and destruction of) the small intrahepatic bile ducts, with accompanying cholestasis, and a typical pattern of serum and secretory autoantibodies reactive predominantly with mitochondrial antigens (AMA; reactivity with PBC-specific antinuclear antibodies (ANA) is also seen). The condition is progressive in most patients, with the development of biliary fibrosis and, ultimately, cirrhosis. The rate of progression to cirrhosis is variable between patients and modified by treatment with UDCA.^7 8^ Criteria defined for the study of the epidemiology of PBC have entered widespread clinical use and underpin inclusion criteria for current trials.^9^ The presence of all three of cholestatic liver biochemistry, AMA or other PBC-specific autoantibody at a titre of >1/40, and diagnostic or supportive liver histology indicates definite PBC; two out of three indicates the presence of probable PBC. In clinical practice, the vast majority of patients are appropriately and confidently diagnosed without a liver biopsy, and in clinical practice the term ’probable PBC' should not be used with patients.^10^ Response to UDCA is variable, and incomplete response is associated with increased risk of death from PBC or need for liver transplantation.^11–18^

### Epidemiology

The epidemiology of PBC has been studied extensively.^19^ PBC meets the criteria for rare disease status (prevalence <50/100 000) in all populations studied.^20^ Data from the largest UK study, in the north-east of England, suggest a prevalence of definite or probable disease of 35/100 000, with an annual incidence of 2–3/100 000.^21 22^ Comparison with other Northern European and North American cohorts suggest these rates are broadly typical.^23–30^ Reported prevalence appears stable following several years of increase. This may reflect a now fully evolved change in diagnostic activity and practice linked to increased awareness of the disease.

PBC prevalence is asymmetrical within the population with markedly higher rates being seen in women than men (the difference is 10-fold).^19^ UK data suggest that PBC is diagnosed at a later stage in men, potentially reflecting perception bias among clinicians.^12^ PBC is also typically a disease of older patients with the median age at diagnosis being 65 years. The dual effects of age and sex mean that PBC can reach a prevalence of as high as 1 in 800 in women over the age of 45 years. PBC is yet to be reliably diagnosed pre-menarche (youngest report is of a girl aged 15 years).^31^ There are potentially important differences in the clinical expression of PBC between men and women and between older and younger patients, although the basic approach to management is the same in all demographic groups.^12^ The impact of ethnicity on presentation is not well described, but there are reports internationally of how ethnicity affects the presentation of autoimmune liver disease, and clinicians should be aware that classical descriptions of disease are frequently derived from Caucasian-only populations.^32–36^

Familial PBC is clearly recognised, with familial rates similar to those seen in other autoimmune conditions. The reported sibling relative risk for PBC is 10.^37^ The relative risk for familial disease is greatest, at 35, for the daughters of mothers with PBC, reflecting in part the disease demographics. Patients with PBC typically have an increased incidence, in both themselves and their families, of other autoimmune diseases (over half of patients with PBC have another autoimmune condition), reflecting shared genetic predisposition (most notably but not exclusively celiac disease, scleroderma, thyroid disease and Sjögren’s).^37–43^

### Aetiology

PBC is an immune-mediated biliary injury. Evidence supports the interaction of immunogenetic and environmental factors in the aetiology of PBC.^3^ The presence of genetic susceptibility is supported by the increased concordance rate in monozygotic twins^44^ and confirmed by the identification of significant numbers of associated genetic loci in Genome Wide Association Studies (GWAS) and other large-scale, high-quality genetic approaches.^45–54^ Identified genetic associations mirror the pattern and nature seen in autoimmune diseases with the combination of a significant number of genetic associations with low OR for risk, typically in genes regulating the magnitude and nature of the immune response.^55^ Study of the genetic basis of PBC remains a research tool and has, as yet, had no impact on clinical practice.^56^ The existence of disease clustering points to environmental triggers, and research has supported both infectious and chemical triggers.^22 28 57–61^ Case–control study approaches, which explore risk history in patients and matched controls, have confirmed cigarette smoking and recurrent urinary tract infections (UTIs) as being strongly associated with PBC; cholestasis and/or pruritus during prior pregnancy is also associated with future diagnosis of PBC.^40 42 43 62^ Other identified (but not confirmed) associations include hair dyeing and perming.^63^ At present there is no consensus as to causality of any environmental association, and the science relating to disease triggering is again a research tool with no immediate clinical relevance in terms of disease prevention in at-risk individuals. It is relevant to document smoking history, recurrent UTIs and pregnancy-related cholestasis; additionally, smoking is associated with more advanced disease at presentation, and guidance regarding cessation is appropriate.

## How is PBC diagnosed?

### Modes and routes of presentation

**Figure 1:**
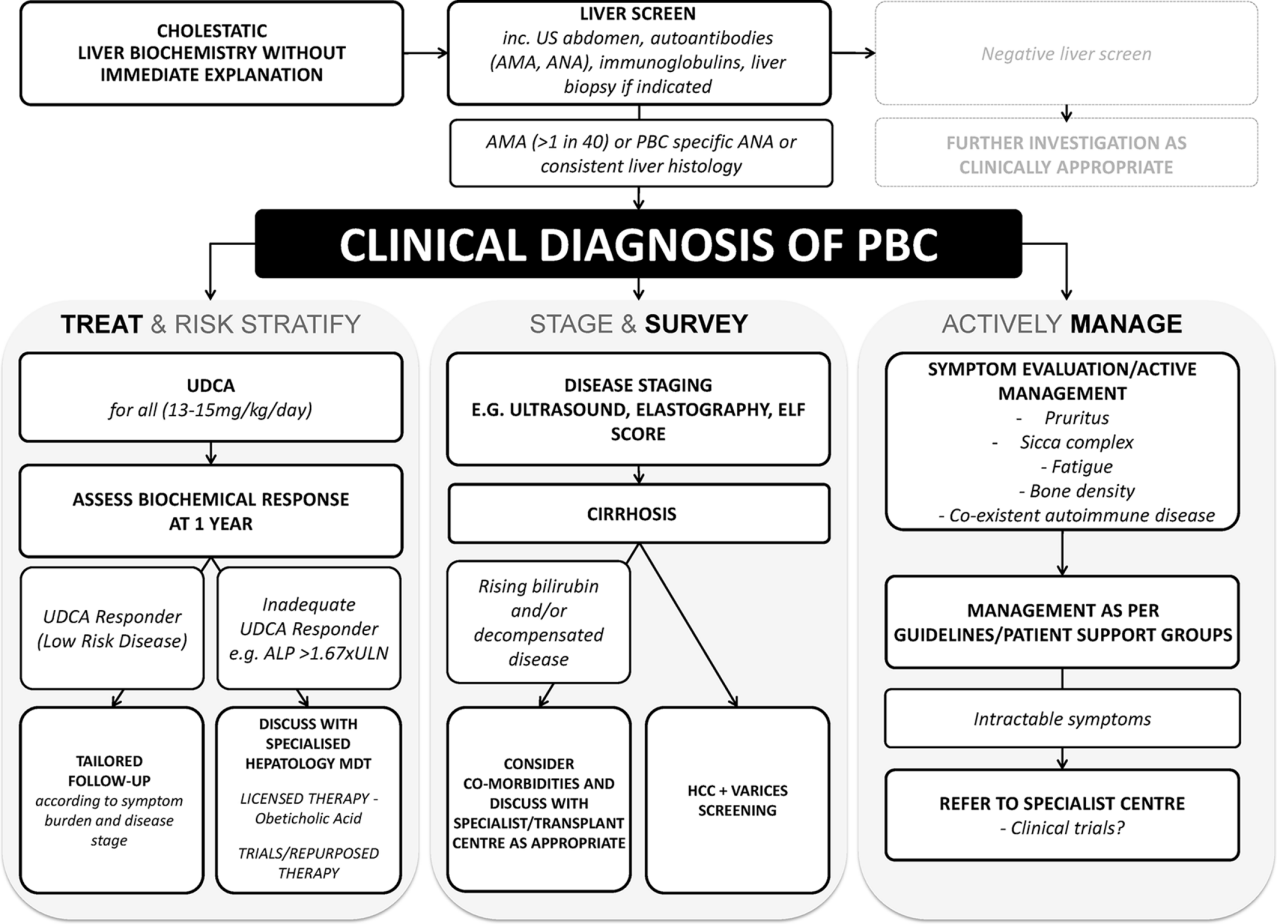
The BSG/UK-PBC consensus care pathway for patients with primary biliary cholangitis (PBC). While care needs always to be personalised to the patient, there are consensus pathways that are important for patients with PBC, which encompass the important ‘pillars’ of care that are believed to provide optimal management of disease and its complications.

Increased awareness of the serological associations of PBC and the widespread use of blood test-based screening in the community has led to an evolution of the mode of presentation of PBC in recent years, away from presentation with clinically overt disease (eg, advanced liver disease)^64^ towards presentation following identification of liver biochemical abnormality on screening^65^(figure 1). Increasing awareness of PBC as a cause of chronic fatigue and pruritus may have led to an increase in diagnosis following symptomatic presentation. Given the efficacy of UDCA treatment in slowing disease progression, it makes sense that early diagnosis may facilitate better outcomes. Treatment failure is seen more commonly in those presenting with cirrhosis and in the ductopenic variant of PBC. Despite awareness of PBC and its target demographic, occasional patients still present with very advanced disease at the point of needing liver transplantation.

### Blood tests

The diagnostic accuracy of the combination of cholestatic serum liver tests and PBC-specific serological markers (>95% for both sensitivity and specificity) means that blood tests lie at the heart of PBC diagnosis.^10^

#### Liver biochemistry

PBC is characterised, in its early stages, by elevation in serum ALP and GGT. Multiple studies on the biochemical response to UDCA therapy demonstrate the value of ALP following therapy as a useful prognostic marker. It is unclear at present whether ALP values are markers of response to other emerging forms of therapy in PBC, but recent Food and Drug Administration (FDA) regulatory review has suggested there is at least reasonable evidence to consider ALP as a surrogate marker of treatment efficacy in PBC. Elevation in bilirubin and fall in serum albumin are features of advanced disease and are also important prognostic markers. Given the more diverse causes of elevations in GGT, to date the utility of GGT determination in patients with PBC has been to confirm a biliary origin of ALP, and not usually to reach a diagnosis or guide therapy. Further studies need to be performed before GGT can replace ALP with regard to diagnosis and treatment, although in the context of classical cholestasis a strong correlation between the two laboratory markers exists.

#### Autoantibodies

PBC is characterised serologically by autoantibodies specific for mitochondrial, nuclear and centromere antigens, some of which are unique to PBC.^66–78^ They are present in ~95% (mitochondrial) and ~30% (nuclear) of patients. Unlike in many other autoimmune diseases, these autoantibodies are, as a result of their sensitivity and specificity, extremely useful in diagnosis and have contributed significantly to the decline in the need for liver biopsy, at least for the purposes of diagnosis. Originally defined in terms of immunofluorescence (IF) patterns (AMA, anti-nuclear dot, anti-nuclear rim, anti-centromere, etc), the identification of the relevant autoantigens (2-oxo-acid dehydrogenase enzymes in the M2 mitochondrial fraction (in particular the E2 component of pyruvate dehydrogenase (PDH)) and the Sp100 and gp210 nuclear membrane proteins, respectively) has allowed the development of ELISA-based diagnostic kits and/or specific immunoblotting. The nature of the approach used for serodiagnosis in PBC (IF vs ELISA) is largely based on local experience and availability, and there is no clear evidence of superiority. IF is operator-dependent and reporting variability can relate to laboratory operator experience. There are also issues around availability and cost of composite tissue block substrates. IF, however, allows subtle autoantibody specificity variations (eg, the non-E2 2-OADC antigens) to be detected. ELISA can have greater sensitivity and is less prone to non-specificity of reactivity resulting from the high levels of polyclonal immunoglobulin M (IgM) seen in PBC. Where PBC-related autoantibodies are detected in the context of an autoantibody profile performed following clinical suspicion of an alternative autoimmune disease, the possibility of undiagnosed PBC must always be considered and liver function tests (LFTs) measured.^77^

A titre of >1 in 40 for any autoantibody linked to PBC is conventionally regarded as being positive.^9^ Caution should be applied in interpreting lower titre autoantibody values because of the risk of non-specific reactivity and thus false positivity. Such findings need to be interpreted in the broader context of clinical presentation and other investigations, including other autoantibody assessment modalities such as ELISA following initial IF assessment. It is common practice in many centres to replicate AMA identified by IF by use of anti-M2 or other ELISA. For routine cases, with clear-cut high-titre reactivity in the primary assay used, there is usually no additional value from a confirmatory second assay.

There is no evidence to suggest that the concentration of AMA above the diagnostic threshold holds any prognostic significance. Repeat measurement is therefore not recommended once a clear-cut diagnosis is established. Additionally, the titre can fall on UDCA therapy and repeated measurement may therefore confuse unnecessarily. There is evidence to suggest that PBC-linked ANA (in particular anti-gp210/anti-nuclear rim antibody) may be associated with more rapidly progressive disease and disease which is less responsive to UDCA therapy.^76 79–82^

The clinical significance of AMA detected in the presence of normal liver biochemistry is currently unclear. Cohort studies from the 1980s showed that such AMA-positive patients with normal LFTs had a high frequency of biliary features of PBC on liver biopsy, and the majority went on to develop classical PBC over prolonged follow-up (although notably not advanced liver disease).^83 84^ More recent large-scale blood donor and population studies have suggested that low titre AMA positivity in the context of normal LFTs is seen in ~0.5% of the population.^85 86^ Whether this apparent increase in AMA positivity reflects false positivity arising, for example, in the context of other chronic inflammatory conditions, increased sensitivity of the modern assays, or a true increase in the prevalence of AMA resulting, for example, from increased environmental triggering with the potential to be followed by a significant increase in PBC incidence over time is unclear. The clinical context of any AMA result is therefore critical and further research is needed in this area. Given the benign prognosis in patients presenting with AMA and normal LFTs even in the historical series, neither biopsy nor use of UDCA therapy is recommended in this group. Follow-up of liver biochemistry in primary care (following initial assessment in the secondary setting) is suggested. Standard advice is for the patient to have serum liver tests repeated annually. If those tests become abnormal, patients should either be re-referred to secondary care (most common practice) or have UDCA commenced if the abnormality in ALP is for longer than 6 months. Additionally, the context of the immunological profile needs to be considered with a lower threshold for intervention in patients with other classic autoimmune diseases such as celiac disease or primary Sjögren’s syndrome.

#### Immunoglobulins

Changes in immunoglobulin G (IgG) and IgM concentrations are seen in patients with PBC. A polyclonal elevation of IgM is characteristic of PBC,^87 88^ with the majority of patients having a non-specific elevation in IgM concentration. In one well-characterised cohort of patients with PBC,^89^ the mean IgM concentration was 2.4 x ULN, and 1.16 x ULN for IgG at baseline. High IgM concentrations (which do not reflect the presence of IgM autoantibody) do not form part of standard diagnostic paradigms but can be useful in making a clinical diagnosis in patients with atypical other features. IgM reduction with UDCA and experimental second-line therapies has been reported, but the prognostic significance of such change has yet to be established and IgM response does not currently feature in any response assessment paradigms.^90 91^ Further research is needed in this area. Elevation of serum IgG can be a feature of the presence of additional autoimmune hepatitis (AIH)-like features in PBC, but is also more commonly reported in AMA-negative series, and is additionally likely equally frequently to be a reflection of advanced fibrosis/cirrhosis generally.

## Recommendation 1

We recommend that any patient with persistently elevated cholestatic liver biochemistry (raised ALP or GGT) without an alternative cause should have autoantibodies checked for anti-mitochondrial (AMA) and anti-nuclear (ANA) reactivity. (Strong; High)

## Recommendation 2

We recommend that the presence of AMA (>1 in 40) or highly PBC-specific ANA, in the appropriate context of cholestatic liver biochemistry, without alternative explanation, is usually sufficient for confidently reaching the diagnosis of PBC. (Strong; High)

## Recommendation 3

We recommend that, for patients in whom the clinical suspicion for PBC is high but classical indicators of disease are discordant (eg, normal liver biochemistry, serology at a low titre), further investigation and review is required prior to establishing a diagnosis of PBC or initiating therapy. (Strong; Moderate)

### Imaging

The role of imaging in the diagnosis of PBC is largely to exclude alternative diagnoses, particularly biliary and infiltrative disease, such that for the vast majority a screening ultrasound suffices. Particular attention to exclusion of primary sclerosing cholangitis (PSC) and its mimics by magnetic resonance cholangiopancreatography (MRCP) is warranted for seronegative patients. Gallstones are a frequent finding in patients with PBC and are typically clinically silent. The over-interpretation of the presence of gallstones in patients of the typical PBC demographic, with the failure to consider PBC as the underlying diagnosis, is a potential reason for delayed diagnosis of PBC. MRCP is typically normal in patients with PBC. Enlargement of the peri-portal lymph nodes is common in PBC (and liver disease generally) and can cause concern about the possible presence of malignancy. Biopsy of such nodes typically shows the presence of reactive/inflammatory changes and the enlargement is thought to be part of the underlying disease process in PBC. Clinical judgement should be used as to whether the rare concern about the possibility of haematological or other forms of malignant disease is sufficient to warrant biopsy exclusion on a case-by-case basis. In end-stage PBC, imaging to screen for the complications of cirrhosis should be routine as for cirrhosis of other aetiology.

### Histological features of PBC

Histopathological evaluation of liver biopsy tissue in PBC can be challenging and interpretation of histological findings needs to be correlated with clinical and immunological features, given the frequent patchy nature of PBC throughout the liver as well as the importance of recognising that, in early stage disease, characteristic features may be absent. As with all liver biopsy interpretation, but notably in the context of biliary disease, adequate biopsy size is essential. The adequacy of any biopsy is of course related to the clinical question, but broadly a liver biopsy should be of large enough size to view a representative amount of parenchyma and number of portal tracts (proposed to be more than 11).^92^ Hallmarks of PBC include destructive granulomatous lymphocytic cholangitis affecting interlobular and septal bile ducts leading to progressive bile duct loss, chronic cholestasis, fibrosis and cirrhosis. Other features that are seen include lymphocytic interface activity, parenchymal necro-inflammation and nodular regenerative hyperplasia.^93 94^ The significance of features such as interface hepatitis is best interpreted through joint clinicopathological discussion. While historically staging of liver disease with biopsy was frequently undertaken, increasingly it is recognised that risk stratification is more relevant to clinical practice, and staging of disease (as is required to determine the need for surveillance of cirrhotic complications) can usually be adequately evaluated non-invasively. Nevertheless, in those for whom biopsy is indicated either because of clinical trial entry or because of concern over diagnosis and/or presence of overlap features, histological stage, presence of ductopenia (>50% bile duct loss) and severity of lymphocytic interface activity are significant predictors of fibrosis progression.^18 95 96^

### Role of liver biopsy and other staging investigations

**Figure 2:**
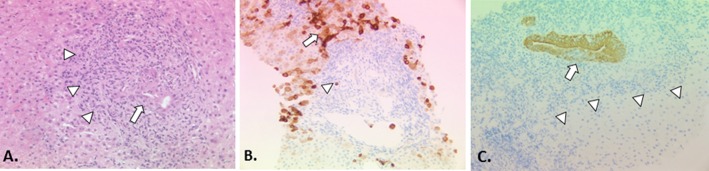
The histopathology of PBC classical staging systems for PBC divide the histological injury of PBC into four stages: florid duct lesions and portal inflammation without interface activity (stage 1), interface hepatitis, ductular proliferation and periportal fibrosis (stage 2), bridging necrosis or bridging fibrosis (stage 3), and cirrhosis (stage 4).^93^ These systems are easy to apply and are quite reproducible. However, their practical utility is limited because of the uneven distribution of diagnostic histological lesions of PBC and different disease stages co-existing at any time.^93^ Furthermore, they incorporate features such as inflammation, which are more appropriately regarded as a manifestation of disease activity (histological ‘grade’) rather than disease progression (histological ‘stage’). A more recent scoring system described by Nakanuma and colleagues sums up individual scores for fibrosis, bile duct loss and severity of chronic cholestasis based on copper-associated protein deposition to assess disease stage and provides a separate system for grading necroinflammatory activity based on cholangitic and hepatitic features.^100^ Similar to the classical staging systems, the Nakanuma staging system correlates well with clinical and laboratory features. Subsequent studies have suggested that the Nakanuma system is more useful than previously described staging systems in predicting adverse outcomes in patients with PBC^101 102^ and may also be helpful in predicting treatment responses.^79^ Another recently described histological scoring system for PBC based on prognostically significant lesions (ie, fibrosis, bile duct loss and lymphocytic interface hepatitis) showed better interobserver agreement and correlation with biochemical abnormalities than traditional scoring systems, but predictive value for adverse outcomes could not be assessed.^103^ Problems with sampling variability apply to all of the histological staging systems that have been described for patients with PBC, which limits the utility of liver biopsy to assess disease severity in routine clinical practice, but they may still have a role in the context of clinical trials where liver biopsies have been used for risk stratification and as a surrogate marker of treatment outcomes. (A) Early PBC is characterised mainly by portal lesions and mild necroinflammatory changes in the acini. Portal tracts may show cholangiocentric granulomatous inflammation composed of lymphocytes, occasionally numerous plasmacytes, and polymorphs including eosinophils. Lymphoid follicles with germinal centres may form. The lymphoid inflammatory infiltrate extends to the biliary epithelium (cholangitis) (arrow), disrupting the basement membrane sometimes leading to bile duct destruction (florid duct lesion). Granulomas, ranging from small collections of histiocytes to easily discerned non-caseating epithelioid granulomas, may be present in portal tracts near damaged bile ducts and less often in the acini. In the progressive lesion of PBC, lymphocytic interface hepatitis may predominate blurring the portal tract boundary and extending into the acinus (arrowheads). Ductular proliferation at the portal-parenchymal interface may be prominent with associated stromal oedema and neutrophilic inflammation. Parenchymal necroinflammatory activity and hepatocellular injury are usually mild. Small and large cell change and hepatocellular regeneration may be seen (H&E, x20). (B) Keratin 7 immunostaining highlights loss of bile ducts (arrowhead indicates a keratin 7-positive bile duct epithelial remnant) leading to chronic cholestasis with features of feathery degeneration, Mallory-Denk bodies, copper-associated protein deposition in periportal/periseptal hepatocytes (cholate stasis), cholestatic rosettes and biliary metaplasia of hepatocytes (arrow) (keratin 7 immunostain, DAB chromagen, x10). (C) Loss of canals of Hering in acinar zone 1 (arrowheads) detected by keratin 19 immunostaining has recently been proposed as an early feature of PBC in the absence of the classic destructive biliary lesions.^104^ Focal intraepithelial inflammation (cholangitis) is noted in the K19-positive interlobular bile duct (arrow) (keratin 19 immunostain, DAB chromagen, x20).

Liver biopsy for the diagnosis of PBC in cases with clear cut autoantibody reactivity and cholestatic liver biochemistry is not recommended as it does not add to the diagnostic accuracy.^10^ It is also not uncommon to see areas of non-involved liver within even cirrhotic liver which, if sampled at biopsy, can confound diagnosis.^97^ Moreover, the yield for diagnostic lesions characteristic of PBC falls to less than 50% in early disease (ie, false-negative biopsies are likely in very early stage disease).^98^ Liver tissue abnormality in PBC can be highly patchy in nature, with reports of all disease stages from 1 to 4 (cirrhosis) being found in the same explanted organ at liver transplantation.^99^ For these reasons, staging biopsy to determine disease progression and establish or exclude the presence of cirrhosis is also not recommended routinely. The existing concept of AIH overlap disease which potentially may benefit from corticosteroid therapy, and the emerging concept of high-risk disease with a low level of response to UDCA and the concomitant need for second-line therapy, mean that liver biopsy may have a value in disease stratification and selection of appropriate additional or second-line therapy in PBC. The precise value and timing of prognostic liver biopsy in PBC remains to be established, as does the role of specific pathological scoring systems. A brief discussion of histological scoring systems^100–104^ which have been used in PBC is included in the legend to figure 2.

Both enhanced liver fibrosis (ELF)^105^ and transient elastography (TE)^106 107^ (eg, FibroScan) have, in cross-sectional studies, shown accuracy in determining disease stage as confirmed by biopsy. There are no data, at the individual patient level, regarding change in these parameters with time and their relationship to change in the disease characteristics. While their use is increasing in clinical practice because of access to ELF testing and/or TE machines, their optimal use is currently a research question and the findings are not, in routine practice, as yet linked into paradigms for location and intensity of patient follow-up. Systematic evaluation of these approaches, together with recently described laboratory parameter-based scoring formulae,^108 109^ in identifying high- and low-risk patients in whom to target enhanced hospital-based monitoring and return to management in primary care, respectively, is warranted.

## Recommendation 4

We recommend all patients with suspected PBC should have a baseline abdominal ultrasound as part of their assessment. (Strong; High)

## Recommendation 5

We recommend liver biopsy is not usually required in the diagnosis of PBC or for monitoring of disease progression unless its use is within the context of clinical trials. (Strong; High)

## Recommendation 6

We recommend there are a range of non-invasive tools to stage and monitor disease progression. There is no consensus as to what is the optimal strategy, but clinicians should be aware of an evolving likely clinical utility. (Strong; Moderate)

## Recommendation 7

We recommend that, in the presence of cholestatic serum liver tests but an absence of diagnostic autoantibodies, the confirmation of PBC requires a liver biopsy. (Strong; Moderate)

## Recommendation 8

We recommend that liver biopsy can be considered if there is a clinical suspicion of co-existing disease (eg, additional injury from non-alcoholic fatty liver disease (NAFLD), viral hepatitis or alcohol use) or the presence of overlapping autoimmune hepatitis, either at diagnosis or during follow-up. (Strong; Moderate)

## What other conditions should be considered in the differential diagnosis of PBC?

The diagnosis of PBC usually causes little confusion because of the specificity and sensitivity of PBC-associated autoantibodies.^110^ Care must be taken in autoantibody-negative disease, with the chief differential being small duct PSC. Other differential diagnoses which should be considered include sarcoid, graft-versus-host disease (in appropriately at-risk individuals), idiopathic ductopenia, drug-induced liver injury and variants of genetic cholestatic syndromes. Care must also be taken with patients with ‘low titre’ AMA because of the danger of autoantibody false positivity in inflammatory conditions, in particular NAFLD, where low level rises in ALP are not infrequent.

## What conditions are associated with PBC?

PBC is principally associated with other autoimmune conditions reflecting shared immunogenetic susceptibility.^19 38^ The strongest association is with Sjögren’s syndrome (most frequently secondary ‘sicca complex’ although primary Sjögren’s syndrome is associated) and the management of the symptoms of sicca complex can be an important part of controlling the overall symptom burden in PBC. The presence of associated thyroid disease (present in up to 25% of patients) or anaemia with an immune/autoimmune aetiology (including pernicious anaemia, autoimmune haemolytic anaemia and celiac disease) should be considered in patients with prominent fatigue. At presentation and follow-up, consideration should be given to testing for these conditions.

Osteoporosis is frequent in PBC, although it is best regarded as a complication of the metabolic changes seen in cholestasis including reduced absorption of fat-soluble vitamins.^111–113^

Recurrent UTIs have been associated with PBC in several epidemiological studies.^62 114^ It is unclear whether the association is a cause or a consequence of PBC, and thus whether aggressively treating UTIs may have any impact on the natural history of PBC. Recurrent UTIs are, however, a potential cause of impaired QoL in PBC and should be effectively managed for this reason.

Despite elevation of cholesterol being a frequent feature in PBC, there is no robust evidence to suggest that ischaemic heart disease or other forms of atherosclerotic disease are seen at increased frequency in the condition.^115–118^ This is likely to reflect the fact that cholesterol elevation is typically high-density lipoprotein (HDL) and lipoprotein X. There is no evidence that statin therapy is associated with increased risk of liver injury and these drugs can be used as would be indicated in patients without PBC.^119–121^ Of relevance to cardiac risk is the observation from case–control epidemiology studies that there is a significant rate of cigarette smoking in PBC.^43 122 123^

## Recommendation 9

We suggest that at baseline it is reasonable to screen all patients with PBC for celiac, thyroid disease and Sjögren’s syndrome. During follow-up, patients should be monitored clinically, and we suggest testing considered for the development of associated autoimmune conditions including celiac, thyroid disease and Sjögren’s syndrome based on clinical need. (Weak; Moderate)

## Recommendation 10

We recommend that there is no substantiated evidence that the hyperlipidaemia of PBC is associated with an elevated cardiac risk, and a patient’s need for lipid-lowering therapy should be evaluated using cardiovascular risk assessment, focusing on identification of patients with PBC and metabolic syndrome (with high cholesterol, low HDL cholesterol and high low-density lipoprotein (LDL) cholesterol levels). There is no added contraindication to the use of HMG-CoA reductase inhibitors in patients with PBC. (Strong; Low)

## What is the natural history of PBC?

PBC is a chronic disease, generally characterised by a slow progression. The clinical course can, however, be highly variable. The majority of patients diagnosed today are asymptomatic at diagnosis and one contemporary series showed that nearly 90% do not have fibrosis when first identified (although others have shown higher rates potentially reflecting case mix).^11 12 26 27^

The initial 10-year follow-up report of asymptomatic disease suggested that 50% of asymptomatic patients became symptomatic over this period of time.^124^ More recent studies with longer follow-up indicate that, although asymptomatic disease tends to progress at a much slower rate than symptomatic disease, survival of both symptomatic and asymptomatic patients with PBC is significantly less than that of the general population.^125 126^ It should be noted, however, that in many earlier studies of symptomatic disease the definition of symptomatic included the presence of features such as jaundice or ascites which would more accurately be regarded as features of advanced disease, the association of which with poor prognosis is unsurprising. One UK study suggested absolute survival was the same regardless of symptoms, although notably the cause of death in those who were asymptomatic was more commonly non-hepatic, and those without symptoms had less severe disease at diagnosis.^65^ There are significant issues with aspects of this historical literature such as age differences in study groups (asymptomatic patients are frequently older at presentation than symptomatic) and the use of symptomatic versus asymptomatic disease as an approach to determining prognosis in practice has largely fallen out of favour. What can be concluded from these and other studies is that PBC is not a benign disease, symptoms are important and frequent, and they should be evaluated more consistently.

Importantly, the presence or absence of cirrhosis taken in isolation is not a highly predictive surrogate marker for risk of death in PBC. A detailed review of liver histology suggests that the presence of a lymphoplasmacytic interface hepatitis is a marker of more rapidly progressive disease^14 96^ and, in another report of four cases, rapidly progressive bile duct loss, even in the absence of cirrhosis, led to liver failure; this is the so-called ‘pre-cirrhotic ductopenic’ variant of PBC, characteristic of early onset symptomatic (pruritus) disease.^127^

It is relevant to appreciate rates of histological progression: Corpechot *et al* described the progression toward cirrhosis in 183 patients treated with UDCA.^96^ The incidence of cirrhosis after 5 years of UDCA treatment was 4% and 59% among patients followed-up from stages 1 (early disease) and 3 (fibrosis), respectively (17% and 76%, respectively after 10 years). The median time for developing cirrhosis from stages 1 and 3 was 25 years and 4 years, respectively. The independent predictive factors of cirrhosis development were serum bilirubin >17 µmol/L, serum albumin <38 g/L and moderate to severe lymphocytic interface hepatitis. Future validation of this observation regarding the importance of interface hepatitis is significant, as are identifying other potential factors. There is no good evidence that the AMA titre correlates with the course of PBC (although it can fall with treatment),^90^ but some groups have suggested specific anti-nuclear antibodies may delineate subtypes of PBC (gp210 with progressive disease and centromere antibodies with portal hypertensive phenotypes).^76 128 129^ This, however, remains to be validated widely.

## Recommendation 11

We recommend all patients with PBC should be offered structured life-long follow-up, recognising that different patients have different disease courses and may require different intensity of follow-up. (Strong; Moderate)

## Stratification of future risk and prognostic scoring systems

PBC is primarily a biliary disease, so when signs of failure of hepatocyte function develop, such as coagulopathy (not corrected by vitamin K) or jaundice, these usually indicate advanced and typically irreversible disease (assuming there is no additional reversible element such as drug injury). There are no symptoms present in patients with purely compensated disease which correlate with outcome (neither fatigue nor pruritus correlate with the severity of disease as judged by serum bilirubin or the Mayo risk score; indeed, pruritus frequently diminishes as the disease progresses).^130^ In one report of development of advanced disease features in 770 patients, ascites was present in 20% and bleeding varices in 10.5% after 10 years of follow-up.^65^ The outlook for patients who develop these complications is worse, and new portal hypertension complicated by ascites is an indicator of poor short-term prognosis. In 143 patients who first developed ascites or peripheral oedema, the mean time to death was 3.1 years.^131^ Once a patient develops jaundice, the natural history of disease in the absence of treatment is more predictable, with the degree of bilirubin elevation correlating strongly with survival. The liver insufficiency phase is characterised by worsening jaundice and is typically pre-terminal: mean survival once the bilirubin is 34 μmol/L is 4 years, and when the bilirubin reaches 102 μmol/L mean survival is only 2 years.^132^ This underpins the concept of thinking about liver transplantation once a patient has a bilirubin level >50 μmol/L and, if otherwise appropriate, listing for liver transplantation when bilirubin is >100 μmol/L. Hepatic encephalopathy (HE), when it occurs, is usually during this phase. As with pruritus, ALP and cholesterol may all paradoxically improve in the pre-terminal stage. The introduction of UDCA therapy, which was demonstrated in early studies to reduce serum bilirubin concentrations, has been shown not to invalidate either the absolute serum bilirubin or the Mayo risk score as prognostic markers.^133^

More sophisticated risk scores designed to predict prognosis in patients with PBC have been developed, and in particular recent approaches to survival analysis have addressed whether simple assessments of biochemical response to treatment, in particular with UDCA, may be useful clinically, and whether variability in such response may underpin the heterogeneity of earlier treatment and outcome studies (table 1).^134^ Stratification by biochemistry has now been reproduced widely across cohorts and is recommended for all patients after 1 year of UDCA therapy. This is in order to identify those high-risk patients who are predicted to have reduced survival and are considered likely to benefit from new disease-modifying therapy trials. In addition to stratification by biochemistry, large-scale studies have been able to confirm clinical observations that age at presentation and gender are also stratifiers of risk. It is currently unclear as to which risk/response criteria are optimal for use in clinical practice. At present it is unclear what the significance is of meeting response criteria prior to therapy and what impact this should have, if any, on the use of UDCA therapy. Conversely, classifying low risk more effectively may have value in reducing secondary care follow-up for many. To that effect, more dynamic linear risk models have been developed, but at this stage we need more information about how to implement these clinically.^108 109^

**Table 1:** Commonly referenced criteria for prognosis based on laboratory indices^134^

Criteria	Treatment response criteria	Sample size	Results
Barcelona criteria^13^	Response to treatment defined by ALP decrease >40% of baseline values or normal levels after 1 year of treatment	192 patients (181 women)	8.9% died or fulfilled criteria for liver transplantation Observed survival higher than that predicted by Mayo model and lower than control population (P<0.001) 61% responded to treatment Survival of responders was significantly higher than that predicted by Mayo model and similar to that estimated for control population (P=0.15)
Paris I criteria^14^	Treatment response defined as: ALP <3xULN and Aspartate transaminase (AST) <2xULN and Bilirubin <1 mg/dL	292 patients	10-year transplant-free survival rate of 90% (95% CI 81% to 95%), compared with 51% (95% CI 38% to 64%) for those who did not (P<0.001)
Paris II criteria^15^	Early stage PBC defined by normal bilirubin and albumin at baseline Response treatment criteria: ALP and AST ≤1.5×ULN with normal bilirubin level	165 patients; average follow-up 7 years	All adverse events observed in non-responders (P<0.001)
Mayo^282^	Response defined as ALP <2 xULN at 6 months	180 patients	After 6 months of UDCA therapy, patients with serum alkaline phosphatase levels less than twice normal (P<0.04) were more likely to respond favourably to treatment over a 2-year period
Mayo^16^	Response defined as ALP <2 xULN at 1 year	73 patients; median 2 years follow-up	Patients with ALP ≥2×ULN had a 2-fold greater likelihood of developing endpoints compared with patients with lower values (23% vs 11%) (P<0.05). Patients with bilirubin >1 mg/dL were four times more likely to develop endpoints compared with those with lower values (33% vs 8%) (P=0.02) Patients with ALP ≤1.67×ULN and bilirubin ≤1 mg/dL had the least likelihood of reaching adverse clinical endpoints
Toronto criteria^18^	ALP <1.67xULN at 2 years of treatment with UDCA	69 patients with follow-up liver biopsy performed approximately 10 years after initial histological diagnosis	Histological progression in stage of fibrosis observed in paired liver biopsies was associated with absence of biochemical response at 2 years: ALP >1.67xULN, P=0.001, OR 12.14, 95% CI 2.69 to 54.74 when defined as an increase in one stage ALP >1.76× ULN, P=0.03, OR 5.07, 95% CI 1.17 to 21.95 when defined as an increase in two stages Ductopenia (>50% loss) predicted histological progression (P=0.012) and biochemical response to UDCA (P=0.002)
Rotterdam criteria^17^	PBC classified as early (pre-treatment bilirubin and albumin values normal), moderately advanced (one level abnormal), or advanced (both values abnormal) Biochemical response defined by normalisation of abnormal bilirubin and/or albumin values	375 patients; median follow-up time 9.7 years	Prognosis for early PBC comparable to Dutch population and better than predicted by Mayo risk score Survival of responders better than that of non-responders (according to Paris and Rotterdam criteria; P<0.001) Prognosis of early PBC comparable for responders and non-responders Prognosis of responders significantly better in those with (moderately) advanced disease

Appreciating the evolution of these risk scores is, however, important in understanding the strengths and weaknesses associated with biochemical risk stratification in PBC. Historically, the Mayo PBC risk score^130^ (age, serum bilirubin and albumin, coagulation time and the presence of fluid retention and/or use of diuretics) was used to predict outcome in late-stage PBC. Similarly, generic scoring systems such as the Model For End-Stage Liver Disease (MELD)^135 136^ or United Kingdom Model for End-Stage Liver Disease (UKELD)^137^ scores are of value once again when the disease is advanced. Parés *et al* assessed the course and survival of patients with PBC treated with UDCA and compared this with the survival predicted by the Mayo model and the estimated survival of a standardised population.^13^ In this study a response to treatment was defined by an ALP decrease greater than 40% of baseline values or normal levels after 1 year of treatment (‘Barcelona criteria’). The observed survival free of transplant was higher than that predicted by the Mayo model, but lower than that of the control Spanish population. Just under two-thirds of patients responded to treatment according to the study definition, and the survival of responders was significantly higher than that predicted by the Mayo model and similar to that estimated for the control population (but only if they were treated at an early stage of disease). In a French study, biochemical response to UDCA was correlated with long-term prognosis in 292 patients.^14^ Those showing ALP <3 ULN, AST <2 ULN and bilirubin ≤17 µmol/L after 1 year of UDCA had a 10-year transplant-free survival rate of 90% compared with 51% (‘Paris I criteria’). A further evolution of this model has focused on early stage disease (‘Paris II criteria’),^15^ while a stratification based on ALP treatment response correlates biochemistry and histological progression (‘Toronto criteria’).^18^ The ‘Rotterdam criteria’ are focused towards liver function/stage, including albumin and bilirubin.^17^ Huet *et al* have used a different approach looking at portal hypertension.^138^ A total of 132 patients had porto-hepatic gradient and biochemical values measured at inclusion and every 2 years. After 2 years of treatment, a decreased or stable porto-hepatic gradient (HR 4.64; 95% CI 2.01 to 10.72) and normalisation of AST level (HR 2.89; 95% CI 1.03 to 8.05) were predictive of better survival on multivariate analysis. Responders (defined as either stable or improved porto-hepatic gradient and normalised AST level at 2 years) had a 15-year survival similar to that of a matched local Canadian population. In keeping with this, Trivedi *et al* in a cohort of over 1000 patients confirmed that the AST:platelet ratio is not only independently associated with outcome, but is additive to classic biochemical stratifiers.^139^ Further refinement of stratification tools has been possible by use of large cohorts, and this has led to two important non-categorical scores: the Global PBC score^109^ and the UK-PBC risk score.^108^ These scoring systems derive from large multicentre cohorts and convey probability of survival on a continuous, as opposed to dichotomous, scale (area under the receiver operator curve >0.9). In addition to being internally validated, the latter in particular has been compared against a healthy age- and sex-matched control population.

It is of course clear that there are varied criteria for evaluating treatment response. In clinical practice the expert group noted that criteria applied to recruitment into clinical trials were the ones seemingly used in wider spread practice at the current time in the UK that is focused around an ALP >1.67 x ULN.

## Recommendation 12

Risk assessment should evaluate disease severity and activity at baseline and on treatment. We recommend a combination of serum liver tests (to identify those with an elevated bilirubin, a platelet count <150 or biochemical disease activity on treatment), imaging (liver ultrasound to identify overt cirrhosis and splenomegaly; TE to identify increased liver stiffness) and recognition of young age at disease onset (<45 years) and male sex. These can all aid risk stratification for patients with PBC. (Strong; Moderate)

## Recommendation 13

To identify those at greatest risk of disease progression, we recommend that all patients have individualised risk stratification using biochemical response indices following 1 year of UDCA therapy. (Strong; High)

## Recommendation 14

Prospective research is required to better evaluate risk stratification tools, but we suggest that UDCA treated patients with an ALP >1.67 x ULN and/or elevated bilirubin <2 x ULN represent a group of high-risk patients in whom there is randomised controlled trial evidence for the addition of second-line therapy. (Weak; Moderate)

## Recommendation 15

It is unclear as to when to repeat risk evaluation in patients stratified to a low-risk group. However, we suggest that all patients with PBC should have annual serum liver tests and documented repeat risk assessment every 3 years. Low-risk patients can be considered to be those without cirrhosis who have an ALP <1.67 x ULN and a normal bilirubin. (Weak; Moderate)

## Recommendation 16

We suggest that care provision should involve a partnership between patients, primary care and hospital-led specialty medicine. Care delivery for an individual patient should encompass patient risk assessment, symptom burden as well as how local services are configured. (Weak; Low)

## Recommendation 17

We suggest that those patients with UDCA non-responsive disease, advanced liver fibrosis/cirrhosis, features of portal hypertension or complex symptoms have disease for which hospital-led care is indicated. We suggest that patients with non-cirrhotic, UDCA-responsive disease without high symptom burden may have disease that, in the context of appropriate service configuration and agreed care pathways, can be led from primary care. (Weak; Low)

## How should patients with PBC be managed?

The care of patients with PBC encompasses many aspects addressing disease progression and symptom control (figure 1).

### Drug therapy to prevent disease progression

#### Ursodeoxycholic acid (UDCA)

Oral UDCA has been studied widely and discussed in depth with regard to its efficacy.^140^ The use of UDCA is recommended for all patients with PBC by AASLD and EASL, as well as in this guideline.^141–151^ A number of other agents have been studied including immunosuppressants, but reproducible and/or consistent evidence of benefit has been universally lacking. Prior studies of failed alternative therapies are not reviewed here in detail.^2^

UDCA normally accounts for about 4% of bile acids, but with pharmacotherapy it becomes the predominant bile acid.^152–154^ Several studies have confirmed a correlation between the degree of bile enrichment and improvement in liver biochemistry. Overall, the data suggest that the optimum dose is 13–15 mg/kg per day, which can be given as a single oral daily dose or in divided doses if tolerability is an issue. When evaluating the UDCA trial data, note must be taken of the treatment dose used because some earlier studies applied lower than optimal treatment paradigms. In PBC, a dose of 13–15 mg/kg/day has been shown to be superior to 5–7 mg/kg/day or 23–25 mg/kg/day. UDCA at the recommended dose is very safe with minimal side effects (weight gain of ~3 kg in the first 12 months, hair loss and, rarely, diarrhoea and flatulence are reported). There are no data to suggest that UDCA is teratogenic. Evidence-based advice over use in pregnancy is lacking, but expert clinical practice generally includes safe use before and during the first trimester; a good safety profile exists from its use in intrahepatic cholestasis of pregnancy.^155 156^

Many studies have attempted to demonstrate clinical efficacy for UDCA and most trials show beneficial effects on biochemical parameters in particular. With such a slow natural history, however, any individual trial in PBC will inevitably lack the power to address end points such as death or liver transplantation. Additional criticism can be made for assuming that every patient benefits equally—that is, identifying and treating more patients with mild disease may be self-fulfilling if those patients were never destined to progress, and historical failure to stratify patients may have confused the literature.

Three large double-blind randomised trials used the same dose of UDCA (13–15 mg/kg/day), and thus the results have been analysed according to an intention-to-treat principle. In two of these a composite ‘treatment failure’ outcome measure was used, and in the third the percentage change in total serum bilirubin over 2 years was used as the primary outcome measure. Few adverse effects of UDCA were reported and the withdrawal rate was less than 20% in all three studies. In two of the three trials a crossover design was adopted, with some patients initially randomised to placebo switching to open-label UDCA after the first 24 months. However, the results were analysed according to intention-to-treat, so that those patients initially randomised to receive placebo and subsequently switched to receive UDCA remained in the placebo group for the purposes of analysis. Ultimately then, this combined analysis of the three trials (548 patients) showed a one-third reduction in the risk of death or transplant for patients with moderate to severe PBC. Subgroup analyses showed that survival free of liver transplantation was significantly improved in medium- and high-risk groups (serum bilirubin 1.4–3.5 or >3.5 mg/dL; P<0.0001 and P<0.03, respectively) and histological stage IV subgroup (P<0.01). One other concern raised was the observation that those patients crossed over to UDCA continued to have a poorer clinical course. A further large trial (151 patients) employed a lower dose (10–12 mg/kg bodyweight daily) and a different preparation of UDCA. After 2 years of treatment no difference in survival was seen, there being eight deaths in those randomised to UDCA and 12 in those randomised to placebo. Prolonged follow-up also showed no survival benefit.

A key factor in the debate over UDCA efficacy is the limitations in the quality of the underlying source trial data with varying, and often inadequate, sample size and duration of several studies, as well as inclusion of data from trials using suboptimal doses. Since not all placebo or non-intervention patients were eventually given UDCA (although a majority were), the evaluation of the non-randomised phases of these trials has biases with implications for the basis for an intention-to-treat analysis. Of the 16 randomised clinical trials evaluating UDCA against placebo, nearly half of the trials had a high risk of bias. In all studies, the administration of UDCA was associated with an improvement in liver biochemistry. An updated Cochrane meta-analysis shows that overt ascites and obvious jaundice are less frequent in patients randomised to UDCA, but there was no difference in the number of patients with bleeding varices or HE. These data suggest that prolonged treatment with UDCA, started at early stages of disease, are likely required to exert a maximal positive effect on the natural course of disease. The meta-analysis that was confined to trials using an appropriate dose of UDCA (>10 mg/kg/day) and with sufficient follow-up (at least 2 years) included a total of 1038 patients (522 who received UDCA and 516 who received placebo). Treatment with UDCA resulted in significant improvement in liver biochemical values. Histological evidence of disease progression was similar for the two treatment groups, but subjects without evidence of fibrosis (stages 1 and 2) who were treated with UDCA had slower disease progression than subjects in the control group. A total of 160 patients who were treated with UDCA and 186 control subjects died or underwent liver transplantation. This difference was significant in a fixed-effect model (OR 0.76; 95% CI, 0.57 to 1.00; P=0.05) but not in a random-effects model (OR 0.77; 95% CI 0.50 to 1.21; P=0.30).

## Recommendation 18

We recommend that oral UDCA at 13–15 mg/kg/day is used as the first-line pharmacotherapy in all patients with PBC. If tolerated, treatment should usually be life-long. (Strong; High)

### Obeticholic acid (OCA)

OCA is a semi-synthetic hydrophobic bile acid analogue that is highly selective for farnesoid X receptor (FXR), having exponential activation potency relative to the endogenous counterpart chenodeoxycholic acid. OCA also induces expression of gut-derived hormones, in particular fibroblast growth factor 19 (FGF-19). The nuclear receptor FXR is a central transcriptional sensor of bile acid metabolic cascades, and FXR is highly expressed in the liver and in enterocytes. The main FXR target gene in the gut is FGF-19, which is an enterokine secreted into the portal blood on bile acid stimulation. FGF-19 reaches the liver where it activates the duo fibroblast growth factor receptor 4 (FGFR4)/beta KLOTHO on the hepatocyte basolateral membrane triggering intracellular pathways that repress cholesterol 7-α-hydroxylase (CYP7A1), which is the rate-limiting enzyme in bile acid synthesis. FXR signalling directly regulates genes involved in bile acid synthesis, secretion, transport, absorption and detoxification; additionally, FXR signalling impacts on inflammation, metabolic regulation and liver fibrosis.^157^

Relevant trial data reflect studies spanning phase II and III drug development. In a phase II randomised double-blind controlled trial of OCA in PBC, the therapeutic efficacy of three doses (10, 25 and 50 mg/day) as add-on therapy to UDCA in a multicentre study restricted to patients having persistent elevations in serum ALP (>1.5 × ULN) was evaluated.^91^ The primary endpoint was a significant reduction in serum ALP from baseline, and was met across all three doses of OCA versus placebo. Moreover, 87%, 69% and 7% of all OCA-treated patients completing therapy achieved a decline in serum ALP of at least 10%, 20% or complete normalisation (vs 14%, 8% and 0% with placebo). In a phase III clinical trial (PBC OCA International Study of Efficacy), patients with PBC with high-risk PBC (prior biochemical non-response according to modified Toronto criteria; ALP >1.67 x ULN and/or elevated total bilirubin <2 x ULN) were evaluated in a randomised placebo-controlled manner.^158^ The primary endpoint during the 12-month double-blind period was attainment of both an ALP value <1.67 × ULN (with a ≥15% reduction from baseline) and a normal serum bilirubin. In an intention-to-treat analysis, biochemical response was met in 10% of the placebo group and in 47% and 46% in the 10 mg and 5–10 mg dose-titrated OCA groups, respectively (P<0.0001 for both). Moreover, the mean decrease in serum ALP from baseline was 39% and 33% in the 10 mg and titrated OCA groups, respectively, versus 5% for patients in the placebo group (P<0.0001 for both). Both OCA groups met predefined secondary endpoints including reduction in serum AST and total serum bilirubin (both OCA groups P<0.001 vs placebo).

Longer-term efficacy of OCA and generalisability to the patient population as a whole needs confirmation in prospective follow-up studies. Survival benefit has yet to be demonstrated and, for that purpose, a long-term randomised trial is currently ongoing. In using OCA, attention is important to assessing the likelihood of benefit, and in those patients with advanced disease dose adjustment is important. More experience is needed in patients with advanced liver disease and, while there were patients with cirrhosis in the pivotal trial of Nevens *et al*,^158^ there were no patients with decompensation. As per the drug label, OCA is dose adjusted to 5 mg weekly initially (with a maximum dose of 10 mg twice weekly) in Child Pugh B or C liver disease. It may well also be prudent when initiating therapy in a patient with Child Pugh A liver disease to also dose adjust in the presence of portal hypertension. Cirrhotic patients, particularly once evidence of portal hypertension exists, should have intensified early safety evaluation (e.g. repeat blood tests monthly at outset) and, in the context of development of decompensation or progression of liver disease, OCA dose adjustment or treatment cessation may be indicated.

Treatment with OCA is associated with a dose-dependent exacerbation in pruritus leading to treatment discontinuation in 1–10% of patients. These observations emphasise the importance of dose titration with or without timely provision of therapy (rifampicin may be preferred, given potential interactions with bile acid sequestrants leading to fecal OCA loss) for symptom control. OCA-treated patients may also exhibit (reversible) alterations in serum lipid levels; specifically, most notably a small decrease in HDL. It is not yet known whether these consequences impact the long-term cardiovascular risk.

NICE has appraised OCA (https://www.nice.org.uk/guidance/ta443) and recommended OCA within its marketing authorisation as an option for treating PBC in combination with UDCA for people whose disease has responded inadequately to UDCA or as monotherapy for those who cannot tolerate UDCA. NICE recommended that clinicians assess the response to OCA after 12 months and that treatment should only continue if there is evidence of clinical benefit.

## Recommendation 19

In patients with an inadequate response to UDCA (or UDCA intolerance) as defined by ALP >1.67 x ULN and/or elevated bilirubin <2 x ULN, the addition of OCA (initial dose 5 mg/day, titrating to 10 mg/day at 6 months if tolerated) has been associated with improvements in biochemical surrogates of disease activity reasonably likely to predict improved outcomes. We recommend, in keeping with the NICE evaluation of OCA, that the addition of OCA for patients with an inadequate response to UDCA, or intolerant of UDCA, is considered. We recommend dose adjustment in patients with advanced liver disease as per the drug label, and careful evaluation of potential benefits and risks, as well as monitoring, in patients with very advanced liver disease (Child Pugh A liver disease with portal hypertension; Child Pugh B and C liver disease). (Strong; Low)

### Off-label therapies

Off-label use of drugs such as budesonide and fibrates has not gained much traction in clinical practice in the UK, in contrast to other countries such as France and Japan. Recommendations for unlicensed therapies in the UK are not presently made pending review and publication of phase III trials as below; this is in keeping with recent guidelines from EASL.^6^

In patients with PBC exhibiting ‘florid’ interface hepatitis on biopsy, there are reports demonstrating the efficacy of budesonide in improving liver histology and biochemistry when used in combination with UDCA. A randomised placebo-controlled trial (n=39) was the first to study budesonide (9 mg/day) as add-on therapy to UDCA in patients with early-stage PBC.^159^ Over the 2-year study period, patients with combination therapy exhibited a significant reduction in serum ALP as well as improvement in liver histology according to the Ludwig classification system. Moreover, in a subsequent 3-year randomised non-blinded study performed in non-cirrhotic PBC patients (n=77), budesonide 6 mg/day plus UDCA (n=46) was associated with a 25% regression in liver fibrosis.^160^ However, despite encouraging results, note must be taken of a high rate of fibrosis progression (an increase of 70%) in patients receiving UDCA monotherapy. In a US open label study of 22 biochemical non-responders (ALP persistently >2 x ULN), only a very minimal additional benefit of budesonide to UDCA was reported, with a significant increase in the Mayo PBC score prognostic index and significant deterioration in bone mineral density; true comparison is challenging, however, because this cohort may have had patients at later stages of disease.^161^ Most notably, a phase III double-blind randomised placebo-controlled trial evaluating UDCA+budesonide vs UDCA +placebo awaits reporting (Eudra CT number 2007-004040-70).

Fibrates exert potent anticholestatic effects through the variable activation of peroxisome proliferator-activated receptors (PPAR), in addition to downregulation of several pathways leading to bile acid synthesis.^162^ It is important for practising clinicians to take clinical note that, while there is long-standing interest regarding these agents in cholestatic liver disease, in the UK drug labelling has documented contraindication to their use in PBC because of concerns over reported hepatotoxicity. Fibrates at high dose inhibit some CYP enzymes, in particular CYP2C9. At therapeutic doses, fibric acid derivatives increase serum alanine transaminase (ALT) and AST levels which may relate to known transcriptional effects on liver transaminase synthesis. For creatinine elevations it may also be that hyperproduction from muscle occurs, and concern over nephrotoxicity requires ongoing investigation and caution. Other adverse effects are recognised: 5–10% of patients, mostly with bezafibrate, develop musculoskeletal pain.

Studies from the 1990s evaluated the use of bezafibrate (400 mg/day) as an adjunctive therapy to UDCA, in which normalisation of serum ALP was reported in ~45% of UDCA non-responders versus ~18% taking placebo.^163^ More recently, a non-blinded prospective randomised controlled study (n=27; 100–120 months of treatment) reported that serum ALP levels were significantly lower following combination therapy (UDCA+bezafibrate) and associated with a trend toward improved overall survival (log rank P=0.057).^164^ Data from an open-label study (n=28) also provide evidence of a significant improvement in itch severity with bezafibrate, wherein all 12 patients who reported itch prior to starting treatment achieved complete or partial symptom resolution.^165^ Moreover, 20 and 24 UDCA non-responders attained a serum ALP reduction >40% within 6 and 12 months, respectively, with combination bezafibrate therapy.

Improvements in serum ALP is also evident through pilot studies using fenofibrate+UDCA combination therapy, with a pooled complete biochemical response rate evident in 69% of patients according to one systematic review and meta-analysis.^166 167^ In a retrospective uncontrolled study, improvements in short-term, liver decompensation-free and transplant-free survival using combination UDCA+fenofibrate therapy independently of liver biochemical changes and across a cohort of 120 prior UDCA non-responders (P<0.001) were described.^168^ However, concern remains about patient ascertainment, and deterioration of some patients with rising bilirubin values.

The biochemical improvements associated with fibric acid derivatives have not been shown to sufficiently alter long-term probability of liver-related death or need for transplantation when stratified according to the UK-PBC risk score,^169^ and may be counterbalanced by a possible negative impact on renal function.^164^ As such, meta-analysis of existing bezafibrate randomised clinical trials show no significant improvement in patient survival compared with UDCA monotherapy,^170^ although liver transplantation and liver-related death were not presented as clinical endpoints. Peer-reviewed results from a phase III clinical trial of bezafibrate in PBC (https://clinicaltrials.gov/ct2/show/NCT01654731) are, however, awaited.

### Symptom management

The symptoms associated with PBC are important and have a significant impact on life quality for patients.^171^ Data from the UK-PBC patient cohort have shown that there is significant variation in management between centres and individual clinicians.^12^ It is hoped that these guidelines will help standardise the approach to symptom management by clinicians. It is our expert opinion that screening for the presence of symptoms by asking about them specifically, followed by offering formal quantification of their impact in patients reporting their presence, can be helpful in understanding the impact on individual patients (approaches can include Likert or visual analogue scales, which are well established for itch particularly and the use of more complex patient-derived measures such as the multi-domain PBC-40 QoL measure^172 173^). Therapies for symptoms should be continuously evaluated rather than on an ad hoc basis, and it is important to re-evaluate symptoms and response to therapy. There is also a risk of recurring symptoms on stopping therapy and most patients require treatment long term. The symptoms of PBC typically do not correlate with disease severity and do not improve with UDCA therapy.^12^

## Recommendation 20

We recommend all patients should be evaluated for the presence of symptoms, particularly fatigue and itch. Clinicians should recognise that the severity of symptoms does not correlate with stage of disease. (Strong; Moderate)

### Pruritus

Pruritus is one of the characteristic cholestatic symptoms in PBC and results in impaired health-related quality of life (HRQoL).^174^ Approximately 80% of patients experience pruritus at some time during the course of their disease.^175^ It can occur at any stage of the disease process, but it is important to note that itch can improve as liver disease worsens.^64^ Patients with the ductopenic variant of PBC have particular problems with itch.^127^ Follow-up of patients and evaluation of change in pruritus and potential side effects is appropriate when changes are made in anti-pruritic therapy.

Bile duct obstruction must be excluded as the cause of pruritus, given the increased risk of gallstone disease in PBC,^176^ although in practice this distinction is rarely problematic. Bile sequestrants are used as first-line therapy but tolerability is often an issue with side effects including bloating and constipation.^177^ Cholestyramine is a non-absorbable resin that may help relieve pruritus. It is important to note that bile sequestrants must be given 2–4 hours before or after other medications (in particular UDCA) as they interfere with intestinal absorption.^178^ Patient education is important here (by clinicians and pharmacists) to avoid drug interactions. There is limited evidence to suggest that UDCA has any effect on pruritus.^12 179^ Colesevelam is a newer, often better tolerated, bile sequestrant which may have a role in management given the better side effect profile compared with cholestyramine. Despite clinicians describing anecdotal experience of benefit, and significant decreases in serum bile acid levels, a recent placebo-controlled trial failed to demonstrate effectiveness.^180^

Rifampicin is a useful second-line agent probably acting through its pregnane X receptor (PXR) agonist function.^181^ Several prospective randomised placebo-controlled trials have shown rifampicin to be effective in the management of cholestatic pruritus.^182–185^ This effect has been confirmed in meta-analyses.^186 187^ There are concerns over potential side effects with rifampicin (including hepatotoxicity and haemolysis), so patients commenced on treatment need regular blood tests.^188^ It is also important to remember that rifampicin affects vitamin K metabolism and can lead to an increase in the international normalised ratio (INR), most notably in icteric patients.^189^ Additionally, appropriate consideration should be given to balancing benefits against risks of antimicrobial resistance.

Opiate antagonists (oral naltrexone and parenteral naloxone) are increasingly used as third-line therapy as they reduce the sensation of itching and scratching activity.^186 190–192^ Naltrexone should be started at a low dose to avoid opiate withdrawal-like reactions in the first few days of treatment.^193^ Long-term tolerability can be an issue, with many patients having ongoing opiate withdrawal-like reactions or reduced threshold to pain.^194 195^

Other drugs which are used empirically in the management of cholestatic itch, typically in patients with pruritus unresponsive to other agents, are selective serotonin reuptake inhibitors (SSRIs; eg, sertraline) and gabapentin. SSRIs presumably act via altering the concentrations of neurotransmitters within the central nervous system. There are some reports of efficacy in the literature, but only a single small placebo-controlled trial.^196^ Side effects of SSRIs include dry mouth and patients should be warned about this. Gabapentin has been suggested as a potential treatment due to the theoretical benefit of increasing the threshold to experience nociception. However, a small trial failed to show benefit over placebo.^197^ Further evaluation of gabapentin may be warranted given the clinical experience. Antihistamines sometimes have a non-speciﬁc anti-pruritic effect which may be due to their sedative properties but are not recommended as specific therapy; they are, however, useful adjuncts for some. Table 2 shows an approach to the treatment of cholestatic pruritus.

**Table 2:** Pragmatic drug approaches to the medical management of cholestatic pruritus in the absence of clinical trial opportunities for patients

Agent	Dose	Additional notes
Cholestyramine	4 g/day to a maximum of 16 g/day as tolerated	Must be given 2–4 hours before or after UDCA (usually give UDCA at night) Pharmacy advice to avoid interactions with concomitant medications Suggest give at breakfast time (an hour before or after eating) if gallbladder in situ; rarely much incremental benefit beyond 8–12 g/day, or tolerance Mixing with orange squash and leaving in fridge overnight improves palatability Gastrointestinal (GI) symptoms: constipation
Rifampicin	300–600 mg/day	Risk of hepatotoxicity – need regular monitoring, start at 150 mg once to twice daily then titrate upwards as per symptoms and lung function test (LFT) monitoring. Maximum 600 mg daily Check LFTs in 2–4 weeks; caution in advanced liver disease; consider vitamin K supplementation if icteric
Gabapentin*	Dose titrate as normal	Dose titrate according to side effects and efficacy
Naltrexone*	50 mg/day (normal maximum dose, although higher doses have been used in the specialist clinic setting)	Start at 12.5 mg/day and titrate slowly to avoid withdrawal symptoms Some patients require an intravenous induction stage
Sertraline*	100 mg/day	Titrate dose to symptoms and as tolerated Needs interaction at the primary/secondary care interface; change over if on alternative antidepressant

*Beyond the routine first- and second-line use of cholestyramine and rifampicin, the choice of other agents is frequently based on an individual clinician’s experience and preference.

Cholestatic pruritus is an area of active research with a number of experimental agents and approaches under development and evaluation. Trials of novel agents, including bile acid reuptake inhibitors and drugs targeting the autotaxin/lysophosphatidic acid pathway recently implicated in cholestatic pruritus, are ongoing or in development.^181 198^ New therapies are likely to emerge in the near future but need evaluation in a clinical setting. Physical approaches, such as nasobiliary drainage,^181 199^ MARS (molecular absorbance recirculating system) and ultraviolet (UV) light therapy are all experimental with case reports/series showing benefit but no formal trial evaluation.^200 201^ UV light therapy is relatively easy to access in comparison to the other treatments. Nasobiliary drainage appears to provide transient relief of itching but requires repeated treatments, is technically complicated and is difficult to tolerate; pancreatitis is recognised as a potentially significant complication. These techniques require further investigation. In extreme situations, temporary relief has been obtained with plasmapheresis or albumin exchange.^202 203^

Liver transplantation for cholestatic pruritus is highly effective in terms of rapid reduction in pruritus severity (frequently within the first 24 hours of transplantation).^204^ Pruritus that is ‘persistent and intractable’ after therapeutic trials is one of the variant syndromes which are indications for liver transplantation according to current guidelines.

## Recommendation 21

We recommend, given the safety profile of bile acid resins, that cholestyramine remains the first-line therapy for pruritus and should be taken separately to UDCA to avoid interaction. (Strong; Low)

## Recommendation 22

We recommend that rifampicin can be a safe and effective second-line therapy for pruritus; prescribers must, however, evaluate the risks and benefits of use and ensure appropriate monitoring for side effects. (Strong; Moderate)

### Fatigue

Although fatigue is not specific to PBC, it is frequently reported by patients (over 50%) and when severe, as it is in 20% of patients, is a significant cause of QoL impairment.^12 171 205–208^ There are peripheral and central components to it, with central fatigue frequently associated with cognitive impairment (poor memory and concentration) which can be mistaken for HE.^209 210^ Fatigue is, with the exception of very endstage patients where it is the norm,^211^ not related to severity of liver disease and is not responsive to UDCA therapy.^12^ The approach to fatigue and its management therefore needs to run, as is the case for pruritus, in parallel with the management of the underlying disease process. Post-transplant patients with PBC typically have ongoing fatigue, and transplant for severe fatigue in the absence of other indications is not appropriate.^12 211^ High quality clinical trials in this area have been limited to date, and there is no licensed therapy. Fatigue in PBC as in other chronic diseases is inherently complex in nature and a structured approach to it is essential if improvement is to be seen.^212^ A structured approach to management, quantifying fatigue and its impacts (through the use of tools such as the PBC-40 QoL measure), addressing contributing and exacerbating factors and supporting patients to cope with its impact has been shown to be effective.^212^ It is important, when addressing fatigue, to identify other disease processes and therapies linked to PBC either directly or indirectly which may be contributing to fatigue. These include other autoimmune conditions such as hypothyroidism or autoimmune anaemias and demography-associated conditions and therapies such as type 2 diabetes and antihypertensive therapy.^38^ The steps to management of fatigue in PBC, which should be taken sequentially, are outlined in table 3. There is no evidence to suggest that exercise is harmful in PBC fatigue. Indeed, there are pilot data to suggest that structured exercise initiated at levels which can be tolerated by fatigued patients may be beneficial.^213^

**Table 3:** Stepwise approach to management of fatigue in patients with PBC

Treat direct contributors	Pruritus Pruritus, particularly at night, is a significant factor in sleep disturbance and secondary fatigue
	Associated autoimmune disease AIH (overlap syndrome), thyroid, celiac disease, pernicious anaemia, autoimmune haemolytic anaemia and Addison’s disease are all linked to PBC, are associated with fatigue and are treatable
	Age-related conditions Diabetes, heart failure and renal failure are common in the typical PBC patient age range, are associated with fatigue and are responsive to treatment
Modify exacerbating processes	Depression This is rarely a primary factor but can exacerbate and treatment may improve overall function
	Autonomic dysfunction Strongly associated with fatigue and in vasomotor forms can cause significant falls; volume repletion and assessment (through 24 hours blood pressure (BP) monitoring and, where appropriate, tilt testing) and adjustment of inappropriate antihypertensive therapy can be helpful
	Sleep disturbance Daytime somnolence can be strongly associated with fatigue; assessment and treatment for obstructive sleep apneoa can be beneficial; case series of the use of modafinil for severe daytime somnolence in PBC with improvement in linked fatigue
Assist with effecting lifestyle adjustments and developing coping mechanisms	Patients need to be advised and supported to develop coping strategies while retaining ownership of the problem. Pacing strategies (using available energy to its best advantage) and timing strategies (fatigue is worse later in the day typically so arranging key tasks for earlier in the day can make them more achievable) are recommended
Support	Fatigue in PBC can reduce life quality. Awareness and understanding from carers is helpful in developing positive patient attitudes and coping strategies

## Recommendation 23

We recommend that alternative causes of fatigue should be sought and treated. (Strong; Moderate)

### Sicca complex

Sicca complex is common in PBC, with symptoms of dry eyes and/or dry mouth frequently seen in patients.^38 214^ Most patients have sicca symptoms rather than primary Sjögren’s syndrome. Other symptoms may include dysphagia and vaginal dryness. Clinicians should specifically enquire about these symptoms. Artificial tears and saliva are often helpful. Pilocarpine or cevimeline (muscarinic receptor agonists) can be used if symptoms are refractory.^215 216^ Patients with severe xerostomia should be given oral hygiene advice to prevent the development of dental caries. Clinicians should also be vigilant of the risk of oral candidiasis in patients with severe xerostomia. Vaginal moisturisers may be helpful but the use of oestrogen creams should be directed in primary care or by a gynaecologist (there are no concerns from a hepatology perspective). Specific guidelines for the management of sicca symptoms and Sjögren’s syndrome should be consulted for further details.^217^ If serological positivity (anti-Ro/La) or extraglandular features are found, evaluation for multisystem disease associated with Sjögrens disease by an expert clinician may be appropriate. Further patients with refractory symptoms should also be referred for specialist management, as evolving new therapies may be available.

### Miscellaneous

Up to one-quarter of patients with PBC have Raynaud’s phenomenon which occurs due to spasmodic arterial contraction in the extremities (usually fingers and toes, but sometimes ears and nose).^38^ Patients should be asked specifically about the classical symptoms of their extremities turning white, then blue and finally red, often associated with pain/burning/tingling when the blood flow returns. Practical measures, such as wearing gloves, using hand warmers and avoiding cold environments, are often all that are needed for mild symptoms. For more marked symptoms, vasodilators such as calcium channel blockers can be used.^218^ Specialist rheumatological advice should be sought for severe symptoms and those at risk of digital ulceration. Approximately 8% of patients with PBC have limited scleroderma (CREST syndrome: Calcinosis, Raynaud’s phenomenon, oEsophageal dysmotility, Sclerodactyly, Telangiectasia).^38^ These symptoms and signs should be sought and, if present, patients should be referred for rheumatology advice. Social isolation, fatigue, anxiety and depression are important predictors of poor perceived QoL in PBC.^219^ Primary care providers should consider assessing patients for features of depression and, where appropriate, a trial of antidepressants may be helpful.

## Recommendation 24

We suggest that patients with symptoms resistant to medical therapy should be referred for specialist management regardless of disease severity. (Weak; Moderate)

### How to manage the clinical needs of a patient with PBC and advanced liver disease

Patients with decompensated liver disease are easy to recognise, allowing institution of appropriate management. It can be more difficult, however, to identify patients with PBC with well-compensated cirrhosis and even liver biopsy can be falsely reassuring due to the patchy nature of disease severity within the liver. There are no defined cut-offs and it is an assessment of relative risk which allows clinicians to decide when a patient requires screening for the complications of cirrhosis.

A practical approaching to identifying cirrhosis in clinical practice is to consider cirrhosis as defined by either confirmation by liver biopsy or on the basis of radiological findings (nodular liver with enlarged spleen) with either a history of complications of liver disease (ascites, variceal bleeding, encephalopathy, pervious bacterial peritonitis) or supportive laboratory findings (low platelets, low albumin, prolonged prothrombin time or INR). Liver stiffness measurement (LSM), assessed by vibration-controlled transient elastography (VCTE), has been shown as one of the best surrogate markers for the detection of cirrhosis or severe fibrosis (ie, bridging fibrosis) in patients with PBC.^107 220^ With increasingly greater access to VCTE now occurring across secondary and tertiary care, many clinicians now use VCTE as an adjunctive tool to help establish whether a patient has significant underlying liver fibrosis/cirrhosis; the results must, however, be interpreted in the clinical context of the patient. In one study, diagnostic thresholds of liver stiffness in discriminating fibrosis stages ≥F1, ≥F2,≥F3 and=F4 were 7.1, 8.8, 10.7 and 16.9 kPa, respectively.^107^ One large collaborative study showed LSM and IQR/median as the two independent criteria of VCTE reliability^221^; EASL-Asociación Latinoamericana para el Estudio del Hígado (ALEH) clinical practice guidelines provide current recommendations on performing VCTE, and clinical interpretation of LSM measurements should account for whether such recommendations are implemented locally—for example, in particular time of VCTE and whether the patient is fasting.^222^

As a result, a combination of clinical markers are used including:

1. Evidence of portal hypertension: thrombocytopenia, splenomegaly and/or varices
2. Histology: biopsy-proven cirrhosis
3. Predictive formulae: for example, Newcastle Varices Score
4. Imaging: ultrasound, cross-sectional evidence of cirrhotic liver/splenomegaly or TE
5. Serum markers of fibrosis: for example, ELF test

In terms of monitoring patients for the development of advanced disease, those who are non-responders to treatment who did not have advanced disease at presentation should have life-long follow-up and annual monitoring for evidence of progression (eg, ultrasound, TE (evidence not clear but accumulating),^106 107 223^ routine blood tests).^105^ Those patients with mild disease and near normal liver biochemistry tests do not require this intensity of follow-up and should have yearly LFTs.

Once a patient with cirrhosis has been identified or the clinical decision has been taken to monitor as if cirrhotic, they should be followed up in accordance with other relevant treatment guidelines for patients with cirrhosis.^4 5 224–226^ Clinical decompensation is, along with bilirubin >50µmol/L, a predictor of adverse outcome in PBC and such patients should be discussed with a hepatologist experienced in managing advanced disease and who is linked to a transplant programme.

## Recommendation 25

We recommend that, in all patients with bilirubin >50 µmol/L (including those treated with UDCA) or evidence of decompensated liver disease, consideration should be made regarding suitability for liver transplantation through discussion with a hepatologist linked to a liver transplant programme. (Strong; High)

### Hepatocellular carcinoma (HCC)

Patients with PBC who have cirrhosis are at increased risk of HCC as in other forms of chronic liver disease.^227 228^ The majority of HCC in patients with PBC occurs in those with cirrhosis, although there are reports of HCC in patients who are non-cirrhotic.^229^ There are some important factors which identify patients as being at increased risk (in addition to cirrhosis). Non-responders to treatment are at greater risk and men are more likely to get HCC than women in PBC (of note given that PBC is much less common in men).^229 230^ Screening should be undertaken in accordance with international guidelines.^224–226^ There is currently an absence of specific UK guidelines. International guidelines currently advise abdominal ultrasound at 6-monthly intervals. Alpha-fetoprotein (AFP) has recently been removed from some international guidelines but is still widely used in clinical practice alongside abdominal ultrasound. The discussion of the health economics of HCC screening in PBC is outside the remit of these guidelines, but note should be taken of increasing locoregional therapies for HCC applicable to patients of all ages.

## Recommendation 26

We recommend that, in patients where cirrhosis is suspected, HCC surveillance should be carried out according to NICE guidelines. (Strong; Moderate)

### Portal hypertension

The incidence of varices in patients with PBC is significant, with approximately one-third of patients with advanced disease developing oesophageal varices over a median of 5.6 years.^5^ At present, relevant guidelines for endoscopic screening do not risk stratify patients. All patients known to have PBC with cirrhosis require endoscopic screening according to prior guidelines.^231–233^ The possibility of occult cirrhosis should also be considered in all patients and factored into decisions about the appropriateness of endoscopic screening. Table 4 shows the various tools available to help identify patients at risk of varices and who might benefit from endoscopic screening. The Baveno VI guidelines use the term ‘compensated advanced chronic liver disease (cACLD)’ to reflect the spectrum of disease in asymptomatic patients and encourage the use of TE in clinical practice.^231^ Patients with a liver stiffness <20 kPa and platelet count >150 000 are at very low risk of having varices that require treatment, although it is acknowledged that patients with PBC were not well represented in the studies to date. Annual assessment using TE and platelet count should be considered. This approach may decrease the number of screening endoscopies required. These tools can be used to help decision making regarding which patients require endoscopic screening, but clinical concern about the presence should always be followed up with endoscopy.

**Table 4:** Tools to help identify patients at risk of varices and who might benefit from endoscopic screening

Tool	Details	Other information
Newcastle Varices in PBC (NVP) Score^283^	Algorithm including ALP, albumin and platelet count with an AUROC for identifying patients with varices of 0.86	Online calculator can be found at www.uk-PBC.com
Mayo Risk Score (not routinely used in the UK)^282^	Independent predictor for the presence of varices; score >4 helps in selecting patients for endoscopic surveillance who are at risk of varices	
Ratio of platelet count to spleen diameter (PC:SD)^284^	Simple tool with a ratio above 909 having a high negative predictive value for the presence of varices	Meta-analysis of 8 studies acknowledged that evidence grading is low and this tool should be incorporated with other assessments of risk^285^
Measurement of portal pressure using the hepatic venous pressure gradient (HVPG)	Most accurate way to risk stratify patients. Clinically significant portal hypertension present when HVPG >10 mm Hg as this is the strongest predictor of the development of varices^286^ and decompensated liver disease^287^	HVPG is invasive and not widely used as a screening tool. It is also important to be aware that HVPG can be inaccurate in PBC due to the possibility of pre-sinusoidal portal hypertension
Transient elastography	LSM correlates with HVPG and identifies patients with significant portal hypertension in various chronic liver diseases^288–290^	

It is important to note that patients with PBC can develop varices even in the absence of established cirrhosis, although in clinical practice pre-sinusoidal varices are relatively unusual.^234–236^ Non-cirrhotic portal hypertension can occur in PBC and the possibility of its presence should be considered in all PBC patients with a GI bleed.

Transjugular intrahepatic portosystemic shunt (TIPS) should be considered for patients with variceal bleeding that fails to respond to endoscopic or pharmacological therapy.^237 238^ Patients with portal hypertension may also develop ascites which should be managed according to current guidelines.^233 239 240^ TIPS has a role in the management of patients with refractory ascites, with a recent randomised controlled trial showing that covered TIPS stents increase the proportion of cirrhotic patients with recurrent ascites surviving transplantation-free for 1 year compared with repeated large volume paracentesis.^241^ Portal hypertension in PBC often has a slowly progressive course and patients may do well with a TIPS.

## Recommendation 27

We recommend that patients with suspected portal hypertension should be screened for gastro-oesophageal varices according to BSG guidelines. (Strong; Moderate)

### Hepatic encephalopathy (HE)

HE can be the cause of significant life quality impairment in patients with advanced disease but is relatively unusual in PBC (and should not be mistaken for the much commoner cognitive impairment associated with fatigue). Where present, HE in PBC characteristically affects older patients. The first-line management is with lactulose (at a dose achieving 2–3 soft stools per day). Some patients may require regular enemas in addition to lactulose. In any patient with HE, it is important to rule out secondary causes such as constipation, dehydration, infection and upper GI bleeding. For patients with refractory encephalopathy, rifaximin is frequently used and is now NICE approved.^242^ Rifaximin is a non-absorbable antibiotic that improves HE, reduces hospital admission rates due to HE and the incidence of recurrent HE.^243–245^ It must be remembered that patients with HE cannot drive.

## Recommendation 28

We recommend that ascites and hepatic encephalopathy should be treated as in standard practice. (Strong; Moderate)

### Transplantation

Liver transplantation is an established and successful procedure that prolongs the life of patients with chronic liver disease and, in certain settings, improves their QoL as well. PBC was among the very early indications for liver transplantation and remains a strong disease indication for surgery.^246^ Liver transplantation, however, remains a challenging procedure and, in most settings, organ availability has a significant impact on determining the precise timing and indications for surgery.

In the UK, patients should have a clear indication for transplantation as well as, usually, a UKELD score of 49 or greater (ie, meet minimal listing criteria based on a biochemical marker of disease severity calculated using the latest bilirubin, INR, creatinine and sodium). Patients with certain variant indications are eligible for listing for transplantation in the absence of an elevated UKELD score, and for patients with PBC this may be relevant (pruritus in particular).^247^

The UKELD score is effective in risk stratification in the context of transplantation and most UK patients are listed for transplantation based on an elevated UKELD score with accompanying liver failure/end-stage liver disease (jaundice, ascites, encephalopathy, variceal bleeding, sarcopenia, HCC) that is not responsive to medical therapy. Fatigue in isolation is not an indication for transplantation. Intractable pruritus unresponsive to medical therapy is an indication for transplantation with good outcomes in terms of pruritus.

In practice it is optimal to ensure patients who may be potentially eligible for transplantation are referred early and/or discussed with centres linked to transplant programmes because this facilitates ready access to transplant services. In practice, in view of the varied nature and timescale for overt decompensation, clinicians should actively consider whether transplantation is the best treatment in any patient with advanced PBC as evidenced by a UKELD score >49, jaundice, portal hypertension or signs of early decompensation (eg, ascites, encephalopathy, sarcopenia).

Recurrent PBC (rPBC) post liver transplant is well recognised but clinically relevant for only a few.^246 248^ It can only be confirmed histologically given that many complications post-transplant (biliary, rejection, vascular) present with an elevated ALP, and serologic features of PBC persist post-transplant so are not additive diagnostically. Protocol liver biopsies are no longer commonly performed and there may be minor changes in liver biochemistry that are not histologically evaluated in patients, therefore the rate of rPBC is an estimate in the literature but is at least ~20% by 10 years. Across studies the reported prevalence rate of rPBC, however, ranges from 0% to 35%. The median time to rPBC ranges between 3 and 5.5 years. Graft loss is possible with rPBC but rare (~1%), and recurrent disease can occur in a second graft. Seemingly, the use of tacrolimus is associated with increased risk of PBC recurrence in the allograft, and some have advocated cyclosporine in patients with PBC as a result^249^; the low clinical relevance of rPBC has, however, meant that practice has not changed. Others have proposed all patients are routinely given UDCA post-transplant, but no consensus exists on this^248^; however, there are recent data supporting UDCA as preventing recurrence. No overwhelming evidence for particular immunosuppressive strategies exist; in particular, there is no evidence to support long-term prednisolone, although in the early post-transplant period a slightly higher rate of acute rejection can be expected.

## Recommendation 29

We recommend that liver transplantation can be an effective treatment for advanced PBC and eligibility should be assessed in line with national guidelines. (Strong; Moderate)

## Recommendation 30

We recommend that pruritus refractory to all medical therapy can be an indication for liver transplantation in highly selected patients regardless of disease stage. (Strong; High)

## Recommendation 31

We recommend that fatigue is not an indication for liver transplantation in patients not meeting established UK liver transplant minimal listing criteria. (Strong; Moderate)

### Fat-soluble vitamin supplementation

While it is rare for patients to develop overt fat-soluble vitamin (vitamin A, D, E, and K) deficiency, this is well described in those with chronic cholestasis, particularly once individuals become jaundiced.^250 251^ Routine measurement of vitamin levels is not usually additive or necessary but, in patients with advanced icteric disease, consideration should be given to oral supplementation of vitamins A, D, E and K using standard preparations.

### Osteoporosis

Osteoporosis affects 20–44% of patients with PBC with the resultant risk of fragility fractures, while the majority of patients have osteopenia.^111^ Risk factors for osteopenic bone disease in PBC include female gender, menopausal status, low body mass index (through the effects of disordered bile acid homeostasis and the pancreatic insufficiency seen in some patients with PBC), older age, advanced disease and chronic cholestasis with resultant vitamin D deficiency.^112^ Patients with PBC also have higher markers of bone resorption (urinary hydroxyproline) and lower markers of bone formation (osteocalcin).^112^

Patients should be given general lifestyle advice to prevent loss of bone density (weight-bearing exercise, smoking cessation, minimising alcohol intake, etc). Falls are seen with increased frequency in PBC due to associated autonomic dysfunction and add to the increased fracture risk presented by osteoporosis.^252^ Patients with a clinical history of falls should be referred to a specialist falls clinic for multidisciplinary assessment, including for the presence of autonomic dysfunction.^252^

All patients with cirrhosis and those with other recognised risk factors (eg, female gender, post-menopausal women, low body mass index, older age) should be assessed for osteoporosis and fracture risk. The FRAX score (the WHO fracture risk assessment tool can be used with or without bone mineral density (BMD) values) or QFracture (BMD values cannot be incorporated into the risk algorithm) should be used to estimate 10-year predicted absolute fracture risk. Following risk assessment with FRAX (without a BMD value) or QFracture, consider measuring BMD with dual-energy X-ray absorptiometry (DXA) in people whose fracture risk is in the region of an intervention threshold and recalculate absolute risk using FRAX with the BMD value.^253^

National guidelines should be referred to for treatment algorithms.^253^ Vitamin D deficiency should be corrected and an adequate dietary intake assured. The use of calcium alongside vitamin D supplementation depends on the adequacy of dietary intake. If a bisphosphonate is required, alendronic acid is usually used first line.^254–257^ Specialist referral should be considered for patients who are unable to tolerate alendronate or risedronate. Treatment options include strontium ranelate, raloxifene, denosumab and teriparatide. There is a widely held view that oral bisphosphonates are unsafe in patients with varices because of the risk of superficial erosion and enhanced bleeding risk. The evidence to support this view is limited. Intravenous bisphosphonates can be used if there is clinical concern. There are limited data regarding the use of hormone replacement therapy and its efficacy in osteoporosis prevention in PBC.^258 259^

## Recommendation 32

We recommend that all patients with PBC should have a risk assessment for osteoporosis. Treatment and follow-up should be according to national guidelines. (Strong; High)

### When should patients be considered for clinical trials?

For many years following the original UDCA trials there has been little or no trials activity in PBC. This is now changing with a number of trials targeting areas of perceived unmet clinic need in the condition,^260 261^ with progress to new licensed second-line therapies such as OCA,^158^ which has FDA and EMA approval, as well as NICE evaluation in the UK. Currently there are trials in three distinct areas and patients should be offered the opportunity to participate if they fall into targeted groups (see https://clinicaltrials.gov/ct2/results?term=primary+biliary+cirrhosis+OR+primary+biliary+cholangitis&Search=Search).

#### High-risk/UDCA-unresponsive disease

The significantly worse clinical outcome seen in patients showing an inadequate response to UDCA has focused interest on trials of enhanced or second-line therapy. Trials currently under development target two aspects of the disease process—namely, the upstream autoimmune response causing initial bile duct injury (typically but not exclusively using second-generation biological-based approaches) and the cycle of cholestatic injury (using second-line bile acid-targeting therapeutics such as FXR agonists that suppress bile acid production and fibrates, which have anecdotal evidence but as yet no randomised placebo-controlled data). The standard approach in current and proposed enhanced disease therapy trials is to target patients not meeting UDCA response criteria at the end of 1 year of treatment at 13–15 mg/kg/day (or who are intolerant of UDCA) and any patient failing to meet this criterion should be considered for participation in ongoing trials of second-line or enhanced therapy.

#### Pruritus resistant to current therapy

Current and proposed trials are targeting resistant pruritus including through breaking of the enterohepatic circulation for bile acids and, it is proposed, targeting the autotoxin pathway. Protocols vary, but typically target patients with significant residual pruritus (defined in terms of severity and/or frequency) following first and established second-line therapy or who are intolerant of current therapy, and should therefore be considered for participation in clinical trials.

#### Fatigue

Trials targeting fatigue are complex, reflecting the nature of the clinical problem.^262^ They form part of the management approach undertaken in specialist centres and patients with severe fatigue should be considered for referral to such centres. Critical for trials of fatigue in PBC is the need to exclude confounding causes, and the systematic approach to addressing such confounding processes outlined elsewhere in these guidelines should be followed before trial participation is considered.

### Management of special populations

#### Variant presentations of PBC

The classical presentation of PBC is in a woman with the combination of cholestatic LFTs and positive AMA, with or without the systemic symptoms of PBC. Important variants are seen in the clinic:

1. *AMA-negative (AMA −ve) PBC:* Approximately 5% of patients with PBC are negative for AMA (or anti PDC-E2/M2 by ELISA), although this figure in practice reflects the assays employed.^263 264^ The majority of these patients will be positive for the PBC-specific nuclear antibodies.^265^ Such antibodies are equivalent to AMA in terms of diagnostic accuracy for PBC and AMA −ve, ANA +ve patients do not need biopsy for diagnostic confirmation. True autoantibody-negative PBC cannot be diagnosed without biopsy. In terms of management, AMA −ve disease should be treated in the same way as AMA +ve. There are data to suggest that ANA +ve patients progress more rapidly.^76^ At present this information does not influence therapy decisions, although this may change in the future with the development of stratified treatment models.
2. *AMA +ve with normal LFTs:* Up to 0.5% of the population in screening studies are found to be AMA +ve with, typically, 50% of those having normal liver biochemistry.^266^ Earlier studies suggested that over prolonged follow-up the majority of patients with AMA and normal LFTs seen in the formal clinical setting went on to develop typical PBC biochemical abnormality and symptoms, although the relevance of this to the broader AMA +ve population is unclear.^84^ Over 18 years of follow-up, however, none developed cirrhosis, needed transplant or died of PBC.^267^ Individuals found to be AMA +ve with normal LFTs should be screened every year for biochemical abnormality development and then treated as for classical PBC if such abnormality is seen. This follow-up can take place in primary care unless there are specific individual factors such as associated autoimmune disease warranting secondary care follow-up. With the background rate of AMA reactivity in blood donors reaching 1 in 200 in some studies, and the rising prevalence of non-alcoholic fatty liver disease, it is also inevitable that AMA reactivity will be identified in patients clinically most likely to have metabolic liver disease. As noted already, such patients may need liver biopsy to identify the dominant liver injury, particularly as an elevation in ALP alone can also be found in patients with NAFLD.
3. *PBC/AIH overlap syndromes:* A small minority of patients with PBC can also have simultaneous AIH features. The management of this group is discussed in detail below.
4. *AMA +ve AIH:* A small minority of patients with AIH are AMA +ve, typically in the context of other AIH-characteristic autoantibodies.^268 269^ Such cases do not usually present a diagnostic challenge because of the presence of a biochemical pattern characteristic of AIH rather than PBC (ALT/AST and IgG elevation rather than ALP and IgM). AMA +ve AIH should be treated as for AMA −ve AIH.

#### Overlap syndromes

A small proportion of patients with PBC also exhibit some or all of the clinical features of AIH. The nature of such ‘overlap’ syndromes, the criteria for their diagnosis and the optimal approach to treatment has been a source of debate for a number of years.^270 271^ PBC/AIH overlap is best not considered as a distinct pathological entity, but rather the reflection of an inherent distribution of clinical features across patient populations presenting with autoimmune liver disease. There have been only a small number of reported putative cases of overlap between PBC and PSC. The critical question is how does the possibility of overlap impact on clinical management? The key distinction is between ‘true’ overlap, where patients are exhibiting definitive features of both conditions, and situations where patients with PBC exhibit features more typically associated with AIH but which fall short of classical diagnostic criteria. The importance of this distinction has been increased by the emerging data suggesting that clinical features which might be superficially suggestive of AIH (elevated serum aminotransferase activity and interface hepatitis on liver biopsy) are in fact also strongly associated with aggressive PBC, predicting both poor outcome and UDCA non-response.

Criteria have been proposed by the Paris group for ‘true’ PBC/AIH overlap,^272^ with two out of three of (a) ALT >5 x ULN, (b) IgG >2 x ULN or positive anti-SMA and (c) liver biopsy with ‘moderate or severe periportal or periseptal lymphocytic piecemeal necrosis (interface hepatitis)’ being suggested as diagnostic of overlap in the context of a PBC diagnosis. Although a diagnosis of PBC/AIH overlap syndrome could theoretically be made without a liver biopsy, uncertainty about establishing the diagnosis means that liver biopsy is still recommended in this situation.^273^

The International Autoimmune Hepatitis Group (IAIHG) does not recommend the use of their criteria, which were developed to identify AIH in isolation not in conjunction with other autoimmune liver conditions, for the diagnosis of overlap.^273^ Based on robust diagnostic criteria, such as the Paris criteria, true PBC/AIH overlap is uncommon (<2% of Caucasian patients),^274^ although there may be differences between different ethnic groups in keeping with ethnic differences in autoimmune disease risk.^36 275^ Where present, however, the outcome may be worse than for classical PBC, with increased risk of the development of complications.^276^ Given the poorer outcome associated with the presence of overlap identified using definitive criteria, treatment augmentation should be considered, with a combination of approaches used to treat both disease elements. Meta-analysis supports the use of combination of immunosuppressive therapy and UDCA in patients with true overlap, but the challenges of disease classification impact directly on the value of such meta-analyses per se.^170^ There is evidence to support the use of budesonide in combination with UDCA, an approach which improves survival in comparison to UDCA monotherapy and is associated with fewer side effects than other immunosuppressive regimes.^170^ Caution must be applied, however, when using budesonide in patients potentially with cirrhosis/portosystemic shunts. Evidence is lacking as to how to approach long-term maintenance therapy in overlap patients stepping down from corticosteroids. It would be reasonable, however, to extrapolate from management regimes for pure AIH with the use of azathioprine (used in conjunction with long-term UDCA).

Patients with parameters diagnostic of PBC or AIH, and associated non-diagnostic features of a second condition, should be treated for the predominant disease in the first instance (in PBC predominant disease with UDCA at 13–15 mg/kg) and the response to therapy assessed,^273^ the rationale being the fact that such AIH-like features are also characteristic of UDCA non-responsive PBC. It is likely that the emerging second-line bile acid therapeutic agents will become the recommended agents for use in such patients following failure to respond to UDCA.

## Recommendation 33

True overlap with AIH is probably rare and we suggest that, when suspected, liver biopsy with expert clinicopathological review is needed to make the diagnosis and guide treatment. (Strong; Moderate)

## Recommendation 34

We suggest that biochemical evidence of marked hepatitic activity (transaminases >5 x ULN), alongside elevated IgG concentrations, are most relevant in considering who should have a liver biopsy. (Weak; Moderate)

## Recommendation 35

We suggest that the presence of severe interface hepatitis in the correct context is usually required to initiate immunosuppression after the risks and benefits of treatment, particularly with corticosteroids, have been discussed with the patient. (Weak; Moderate)

### Pregnancy and PBC

While most patients are diagnosed at an age when pregnancy is not a relevant consideration, a significant minority of patients with PBC are women of reproductive age. In this younger age range of PBC, pregnancy may either be a reason for diagnosis (failure of resolution of obstetric cholestasis) or may be complicated by worsening pruritus. Significant medical risks are infrequent but can be relevant if patients have cirrhosis and portal hypertension. In this setting, management is no different from any other aetiology of cirrhosis (eg, gastroscopy if concern over portal hypertension; exclusion of splenic artery aneurysm by ultrasound).

PBC specific experience is limited to case series, but expert clinical opinion is that UDCA is safe during conception, pregnancy and post-partum.^277^ Additionally, cholestyramine and rifampicin (second trimester onwards) are considered safe in pregnancy, although the data are limited.^155 278^ Rarely, itch during pregnancy becomes unbearable and plasmapheresis may help.^279^ In those with notable cholestasis, fat-soluble vitamin deficiency should be avoided. Post-partum cholestatic flares have been described and clinical follow-up in the post-partum period is important.

Pre-pregnancy counselling should be pragmatic; recognition that in those with a marked ductopenic variant of PBC, disease progression from intense added cholestasis during pregnancy does need consideration. Similarly, patients with portal hypertension have the greatest risks associated with pregnancy and should be appropriately counselled. Variceal bleeding can occur in patients with cirrhosis of any aetiology as a consequence of pregnancy-related increase in portal pressure. Such patients should be electively endoscoped in the second trimester and managed appropriately. Pregnant patients with PBC should be screened for anti-Ro and anti-La antibodies, as their presence would change obstetric practice regarding fetal screening for bradycardia.

## Recommendation 36

Pregnancy is typically well tolerated in non-cirrhotic patients with PBC, but pruritus can be exacerbated. We recommend specialist advice as appropriate for pregnant patients with PBC, including guidance over use of UDCA and treatment of pruritus. While data regarding UDCA in pregnancy are limited, we recommend that expert practice is to continue use peri-conception, peri-partum and post-partum. (Strong; Moderate)

## Recommendation 37

Pregnancy in patients with cirrhosis carries a higher risk of maternal and fetal complications. We recommend patients with features to suggest advanced liver disease have pre-conception counselling and subsequent interdisciplinary specialist monitoring during pregnancy. (Strong; Moderate).

### Familial screening

Awareness of the increased risk of PBC seen in the first-degree relatives of patients with PBC and the role played by genetic factors in disease pathogenesis can give rise to anxiety among patients with regard to the risk that their relatives run of developing the condition. There can be particular concern in the daughters of mothers with PBC because of the female predominance of the disease. Screening for any disease must balance any benefit resulting from earlier diagnosis of the condition against the individual and healthcare costs associated with the screening activity. In the case of PBC, the sibling relative risk is 10 (siblings of a patient with PBC have a 10.5-fold higher risk of developing the disease than age- and sex-matched community controls), while the relative risk rises to 35 for the daughters of patients with PBC.^37^ The prevalence of PBC in the UK population has been estimated as being 350/million (700/million women), giving a prevalence for PBC among the daughters of mothers with the disease of ~2%. Given the low likelihood of screening being positive, the lack of time-dependent therapy where early diagnosis materially alters the nature of therapy, and anecdotal reports of people being screened for PBC subsequently having difficulty getting life and travel insurance, formal screening for PBC in relatives of patients is not recommended. Patient anxiety, however, needs to be taken into account and may be a relevant factor to consider in deciding about ad hoc familial screening.

## Recommendation 38

We recommend that the relatives of patients with PBC do not need to be routinely screened for PBC. (Strong; Moderate).

### Patient support and patient education

NICE recommends in the guideline ‘Patient Experience in Adult NHS Services: Improving the Experience of Care for People Using Adult NHS services’ that clear, consistent, evidence-based, tailored information is available to patients throughout all stages of their care.^225^ In PBC, evidence exists from qualitative research to show that factors such as knowledge, information, consistency, a positive approach, simplification and repetition lead to a positive diagnosis experience.^280^ Findings were used to develop a patient information DVD with expert clinicians describing PBC and patients talking about their experiences. This DVD allows consistent evidence-based information to be provided to patients. It is available to patients and professionals by contacting the patient charity LIVErNORTH (info@livernorth.org.uk; http://www.livernorth.org.uk/pages/factsheet.htm#DVD). Answers to some frequently encountered concerns over PBC care are shown in box 1 and table 5. NICE (https://www.nice.org.uk) also recommends that patients are given both oral and written information. Leaflets are available from a number of national and local patient support groups and are written by clinicians with patient input (The British Liver Trust, The PBC Foundation and LIVErNorth). Leaflets can be obtained by contacting UK-PBC via the website (http://www.uk-pbc.com/). Leaflets should be made readily available to patients.

Box 1 **Situations to consider consultation with a centre hosting a specialist hepatology programme**

- Disease unresponsive or under-responsive to UDCA
- Age at diagnosis: young patients with PBC are at higher risk of progressive disease
- Approaching need for consideration of liver transplantation
- Patients who may require transplant who need complex non-liver surgery
- HCC complicating PBC
- Overlap syndromes
- Intractable symptoms unresponsive to conventional therapy
- Complex therapeutic questions—for example, where other drugs with potential impact are being considered for intercurrent disease (eg, biologicals for rheumatological disease)

**Table 5:** Frequently encountered clinical questions

Frequently asked questions	Current opinion
Can patients with PBC take exercise?	It is perfectly safe to take exercise with PBC and in fact there is some pilot trial evidence that exercise therapy is helpful for the treatment of fatigue.^213^ Patients with PBC frequently lack confidence to undertake exercise so support can be useful^291^
Do patients with PBC need to follow a specific diet?	Expert opinion is that the vast majority of patients with early stage disease, and who are not overtly cholestatic, have no dietary problems and can eat a normal healthy diet. Where patients have cholestasis or one of the associated malabsorption syndromes, fat malabsorption can be an issue which can lead to nutritional problems.^292^ In this group, fat-soluble vitamin deficiency should be considered^251 293^
Should patients with PBC give up smoking?	General advice for health is to stop smoking. There is, however, also specific evidence in PBC to suggest that smoking is more prevalent,^294^ and may be associated with more aggressive disease.^122^ There is therefore a specific rationale for patients with PBC to avoid smoking
Can patients with PBC drink alcohol?	There is no evidence to support an association between either the development of PBC or disease severity, and expert opinion is therefore that there is no reason why patients with PBC cannot drink alcohol within accepted safe limits. Patients with advanced liver disease are however advised to abstain from alcohol
Are any drugs contraindicated in PBC?	As with any liver disease, expert opinion is that caution must always be applied in therapeutics; however, there are no specific concerns regarding drug toxicity in PBC per se
Is PBC associated with cancer risk?	This issue has been extensively looked at^295^ and the only malignancy associated with PBC is HCC in patients with advanced disease^296^ (with a particularly increased risk in UDCA non-responding patients and in male patients.^297^ Previous concerns regarding breast cancer risk have not been substantiated in well-designed studies^298^
Is PBC inherited?	Daughters in particular of patients with PBC show a slightly increased risk of the disease, but this does not represent Mendelian inheritance.^37^ It is thought to represent either shared immunogenetic susceptibility or, potentially, shared exposure to environmental triggers. The lifetime risk of the daughter of a patient with PBC in the UK developing PBC is less than 1%, and on this basis screening is not routinely recommended
Is cardiac risk increased in PBC?	This has been extensively explored and there is no robust evidence to suggest that cardiac atherosclerotic risk is increased in PBC,^299 300^ despite the elevations in cholesterol seen in the disease. Patients with PBC do, however, have a normal level of cardiac risk and appropriate cardiac preventative screening and intervention is recommended. Differential cholesterol assessment is necessary because of the HDL hypercholesterolaemia of the condition and the smoking association is key
Is PBC transmissible to others?	No; although infectious agents have been postulated as triggers for disease, there is no evidence that shared exposure triggers disease and patients should be advised and reassured

A number of online resources are available for patients. Recommended web sites include:

https://www.nhs.uk/conditions/primary-biliary-cirrhosis-pbc/.

http://www.uk-pbc.com/ (UK-PBC).

http://www.britishlivertrust.org.uk/ (The British Liver Trust).

http://www.pbcfoundation.org.uk/ (The PBC Foundation).

http://www.livernorth.org.uk/index.htm (LIVErNorth).

http://www.liver4life.org.uk/ (Liver4Life).

The use of international web sites by patients is not recommended as the clinical practice described may differ from that in the UK, causing confusion.

Fatigue has been shown to be the symptom with the biggest impact on patients. Fatigued patients perceive a poor QoL compared with controls and their levels of social engagement are lower.^171 219^ Very little is written in relation to social isolation and improving support mechanisms in PBC, but there are a number of telephone helplines and patient support groups that offer free qualified peer support to patients. It is recommended (based on expert opinion) that details of helplines can be suggested to patients who may be at risk of social isolation. Information can be found on the following web pages:

- http://www.pbcfoundation.org.uk/Home/CMSPageView/532 (The PBC Foundation)
- http://www.livernorth.org.uk/pages/contact.htm (LIVErNorth)
- http://www.liver4life.org.uk/helpline.html (Liver4Life)

There may be scope for psychological approaches, such as cognitive behavioural therapy, to be used to support patients with PBC. Such approaches have been found to be effective in other chronic conditions for managing distress resulting from debilitating symptoms. Blackburn *et al* explored the psychological impact of fatigue in PBC using semi-structured interviews and validated assessment tools for psychological symptoms. Patients with PBC who report high levels of fatigue were found to be more vulnerable to emotional distress and are more likely to perceive that their QoL has been negatively affected.^281^ We therefore advise that a patient with profound psychological distress associated with fatigue should be referred to appropriate psychological services for assessment.

## Recommendation 39

We recommend that patients with PBC should be offered the chance to seek support from patient support groups. (Strong; Moderate).

## Service standards/audit recommendations for PBC

Opportunities exist to implement the BSG/UK-PBC PBC guidelines into clinical practice through audit of current and future clinical care. We propose that the following service standards and targets be adopted by clinical teams caring for patients with PBC, with the goal being improved and more effective and uniform care for patients with PBC:

1. To exclude alternative aetiologies for cholestasis, all patients with suspected PBC should have an abdominal ultrasound as part of their baseline assessment (standard 90%).
2. All patients should be offered therapy with UDCA. UDCA at 13–15 mg/kg/day is recommended for first-line use in all patients with PBC (standard 90% of patients receiving therapy at adequate dose or documented to be intolerant).
3. To facilitate the identification of patients at risk of progressive disease, individualised risk stratification using biochemical response indices is recommended following 1 year of UDCA therapy (standard 80% of patients receiving UDCA therapy to have their response status recorded in the notes and the criteria used recorded).
4. To highlight the impact on QoL and to ensure appropriate investigation and treatment, all patients should be evaluated for the presence of symptoms, particularly fatigue and itch (standard 90% of patients have the presence/absence of fatigue and pruritus recorded in the notes in the last year).
5. To maximise the opportunity for all patients to be considered in a timely way for liver transplantation, all patients with a bilirubin >50 µmol/L or evidence of decompensated liver disease should be discussed with a hepatologist linked to a transplant programme (; s tandard 90 % documentation that discussion has taken place within 3 months of the bilirubin exceeding 50 µmol/ L and the actions taken recorded)
6. To optimise prevention of osteoporotic bone fractures, all patients with PBC should have a risk assessment for osteoporosis. Treatment and follow-up should be according to national guidelines (standard 80% assessment within the last 5 years).
7. To ensure timely but considered diagnosis and treatment, overlap with AIH should be recognised as rare and, when suspected, liver biopsy with expert clinicopathological assessment is recommended to make the diagnosis (standard 90% of patients in whom the diagnosis of overlap is made having had liver biopsy confirmation and the clinicopathological assessment discussion noted).

## Recommendation 40

We recommend that clinicians caring for patients with PBC should consider introducing clinical audit tools to document and improve the quality of care delivered to patients. (Strong; Low)

## References

[1] Tham TC, Gleeson D, Greenfield SM, et al British Society of Gastroenterology policy and processes for the development of guidelines. Gut 2015;64:1184–5. 10.1136/gutjnl-2015-309164 25666194

[2] Selmi C, Bowlus CL, Gershwin ME, et al Primary biliary cirrhosis. Lancet 2011;377:1600–9. 10.1016/S0140-6736(10)61965-4 21529926

[3] Hirschfield GM, Gershwin ME The immunobiology and pathophysiology of primary biliary cirrhosis. Annu Rev Pathol 2013;8:303–30. 10.1146/annurev-pathol-020712-164014 23347352

[4] European Association for the Study of the Liver. EASL Clinical Practice Guidelines: Management of cholestatic liver diseases. J Hepatol 2009;51:237–67. 10.1016/j.jhep.2009.04.009 19501929

[5] Lindor KD, Gershwin ME, Poupon R, et al Primary biliary cirrhosis. Hepatology 2009;50:291–308. 10.1002/hep.22906 19554543

[6] European Association for the Study of the Liver. EASL Clinical Practice Guidelines: The diagnosis and management of patients with primary biliary cholangitis. J Hepatol 2017;67:145–72. 10.1016/j.jhep.2017.03.022 28427765

[7] Beuers U β1 integrin is a long-sought sensor for tauroursodeoxycholic acid. Hepatology 2013;57:867–9. 10.1002/hep.26228 23456677

[8] Lindor K Ursodeoxycholic acid for the treatment of primary biliary cirrhosis. N Engl J Med 2007;357:1524–9. 10.1056/NEJMct074694 17928600

[9] Myszor M, James OF The epidemiology of primary biliary cirrhosis in north-east England: an increasingly common disease? Q J Med 1990;75:377–85. 2385742

[10] Zein CO, Angulo P, Lindor KD When is liver biopsy needed in the diagnosis of primary biliary cirrhosis? Clin Gastroenterol Hepatol 2003;1:89–95. 10.1053/cgh.2003.50014 15017500

[11] Lammers WJ, van Buuren HR, Hirschfield GM, et al Levels of alkaline phosphatase and bilirubin are surrogate end points of outcomes of patients with primary biliary cirrhosis: an international follow-up study. Gastroenterology 2014;147:1338–49. 10.1053/j.gastro.2014.08.029 25160979

[12] Carbone M, Mells GF, Pells G, et al Sex and age are determinants of the clinical phenotype of primary biliary cirrhosis and response to ursodeoxycholic acid. Gastroenterology 2013;144:560–9. 10.1053/j.gastro.2012.12.005 23246637

[13] Parés A, Caballería L, Rodés J Excellent long-term survival in patients with primary biliary cirrhosis and biochemical response to ursodeoxycholic acid. Gastroenterology 2006;130:715–20. 10.1053/j.gastro.2005.12.029 16530513

[14] Corpechot C, Abenavoli L, Rabahi N, et al Biochemical response to ursodeoxycholic acid and long-term prognosis in primary biliary cirrhosis. Hepatology 2008;48:871–7. 10.1002/hep.22428 18752324

[15] Corpechot C, Chazouillères O, Poupon R Early primary biliary cirrhosis: biochemical response to treatment and prediction of long-term outcome. J Hepatol 2011;55:1361–7. 10.1016/j.jhep.2011.02.031 21703194

[16] Momah N, Silveira MG, Jorgensen R, et al Optimizing biochemical markers as endpoints for clinical trials in primary biliary cirrhosis. Liver Int 2012;32:790–5. 10.1111/j.1478-3231.2011.02678.x 22136310

[17] Kuiper EM, Hansen BE, de Vries RA, et al Improved prognosis of patients with primary biliary cirrhosis that have a biochemical response to ursodeoxycholic acid. Gastroenterology 2009;136:1281–7. 10.1053/j.gastro.2009.01.003 19208346

[18] Kumagi T, Guindi M, Fischer SE, et al Baseline ductopenia and treatment response predict long-term histological progression in primary biliary cirrhosis. Am J Gastroenterol 2010;105:2186–94. 10.1038/ajg.2010.216 20502446

[19] Griffiths L, Dyson JK, Jones DE The new epidemiology of primary biliary cirrhosis. Semin Liver Dis 2014;34:318–28. 10.1055/s-0034-1383730 25057954

[20] Boonstra K, Beuers U, Ponsioen CY Epidemiology of primary sclerosing cholangitis and primary biliary cirrhosis: a systematic review. J Hepatol 2012;56:1181–8. 10.1016/j.jhep.2011.10.025 22245904

[21] James OFW, Bhopal R, Howel D, et al Primary biliary cirrhosis once rare, now common in the UK?. Hepatology 1999;30:390–4. 10421645 10.1002/hep.510300213

[22] McNally RJ, James PW, Ducker S, et al No rise in incidence but geographical heterogeneity in the occurrence of primary biliary cirrhosis in North East England. Am J Epidemiol 2014;179:492–8. 10.1093/aje/kwt308 24401563 PMC3908630

[23] Kim WR, Lindor KD, Locke GR, et al Epidemiology and natural history of primary biliary cirrhosis in a US community. Gastroenterology 2000;119:1631–6. 10.1053/gast.2000.20197 11113084

[24] Sood S, Gow PJ, Christie JM, et al Epidemiology of primary biliary cirrhosis in Victoria, Australia: high prevalence in migrant populations. Gastroenterology 2004;127:470–5. 10.1053/j.gastro.2004.04.064 15300579

[25] Rautiainen H, Salomaa V, Niemelå S, et al Prevalence and incidence of primary biliary cirrhosis are increasing in Finland. Scand J Gastroenterol 2007;42:1347–53. 10.1080/00365520701396034 17918011

[26] Baldursdottir TR, Bergmann OM, Jonasson JG, et al The epidemiology and natural history of primary biliary cirrhosis: a nationwide population-based study. Eur J Gastroenterol Hepatol 2012;24:824–30. 10.1097/MEG.0b013e328353753d 22562114

[27] Pla X, Vergara M, Gil M, et al Incidence, prevalence and clinical course of primary biliary cirrhosis in a Spanish community. Eur J Gastroenterol Hepatol 2007;19:859–64. 10.1097/MEG.0b013e328277594a 17873609

[28] Koulentaki M, Mantaka A, Sifaki-Pistolla D, et al Geoepidemiology and space-time analysis of primary biliary cirrhosis in Crete, Greece. Liver Int 2014;34:e200–7. 10.1111/liv.12479 24502439

[29] Boonstra K, Kunst AE, Stadhouders PH, et al Rising incidence and prevalence of primary biliary cirrhosis: a large population-based study. Liver Int 2014;34:e31–8. 10.1111/liv.12434 24387641

[30] Myers RP, Shaheen AA, Fong A, et al Epidemiology and natural history of primary biliary cirrhosis in a Canadian health region: a population-based study. Hepatology 2009;50:1884–92. 10.1002/hep.23210 19821525

[31] Dahlan Y, Smith L, Simmonds D, et al Pediatric-onset primary biliary cirrhosis. Gastroenterology 2003;125:1476–9. 10.1016/j.gastro.2003.08.022 14598264

[32] Arbour L, Rupps R, Field L, et al Characteristics of primary biliary cirrhosis in British Columbia’s First Nations population. Can J Gastroenterol 2005;19:305–10. 10.1155/2005/203028 15915245

[33] Liu H, Liu Y, Wang L, et al Prevalence of primary biliary cirrhosis in adults referring hospital for annual health check-up in Southern China. BMC Gastroenterol 2010;10:100 10.1186/1471-230X-10-100 20815889 PMC2944334

[34] Harada K, Hirohara J, Ueno Y, et al Incidence of and risk factors for hepatocellular carcinoma in primary biliary cirrhosis: national data from Japan. Hepatology 2013;57:1942–9. 10.1002/hep.26176 23197466

[35] Peters MG, Di Bisceglie AM, Kowdley KV, et al Differences between Caucasian, African American, and Hispanic patients with primary biliary cirrhosis in the United States. Hepatology 2007;46:769–75. 10.1002/hep.21759 17654740 PMC4167731

[36] Levy C, Naik J, Giordano C, et al Hispanics with primary biliary cirrhosis are more likely to have features of autoimmune hepatitis and reduced response to ursodeoxycholic acid than non-Hispanics. Clin Gastroenterol Hepatol 2014;12:1398–405. 10.1016/j.cgh.2013.12.010 24361417

[37] Jones DE, Watt FE, Metcalf JV, et al Familial primary biliary cirrhosis reassessed: a geographically-based population study. J Hepatol 1999;30:402–7. 10.1016/S0168-8278(99)80097-X 10190721

[38] Watt FE, James OFW, Jones DEJ Patterns of autoimmunity in PBC patients and their families. QJM 2004;97:397–406. 15208427 10.1093/qjmed/hch078

[39] Parikh-Patel A, Gold EB, Worman H, et al Risk factors for primary biliary cirrhosis in a cohort of patients from the United States. Hepatology 2001;33:16–21. 10.1053/jhep.2001.21165 11124815

[40] Gershwin ME, Selmi C, Worman HJ, et al Risk factors and comorbidities in primary biliary cirrhosis: a controlled interview-based study of 1032 patients. Hepatology 2005;42:1194–202. 10.1002/hep.20907 16250040 PMC3150736

[41] Lazaridis KN, Juran BD, Boe GM, et al Increased prevalence of antimitochondrial antibodies in first-degree relatives of patients with primary biliary cirrhosis. Hepatology 2007;46:785–92. 10.1002/hep.21749 17680647

[42] Prince MI, Ducker SJ, James OF Case-control studies of risk factors for primary biliary cirrhosis in two United Kingdom populations. Gut 2010;59:508–512. 10.1136/gut.2009.184218 20332522

[43] Corpechot C, Chrétien Y, Chazouillères O, et al Demographic, lifestyle, medical and familial factors associated with primary biliary cirrhosis. J Hepatol 2010;53:162–9. 10.1016/j.jhep.2010.02.019 20471130

[44] Selmi C, Mayo MJ, Bach N, et al Primary biliary cirrhosis in monozygotic and dizygotic twins: genetics, epigenetics, and environment. Gastroenterology 2004;127:485–92. 10.1053/j.gastro.2004.05.005 15300581

[45] Hirschfield GM, Liu X, Xu C, et al Primary biliary cirrhosis associated with HLA, IL12A, and IL12RB2 variants. N Engl J Med 2009;360:2544–55. 10.1056/NEJMoa0810440 19458352 PMC2857316

[46] Mells GF, Floyd JA, Morley KI, et al Genome-wide association study identifies 12 new susceptibility loci for primary biliary cirrhosis. Nat Genet 2011;43:329–32. 10.1038/ng.789 21399635 PMC3071550

[47] Hirschfield GM, Liu X, Han Y, et al Variants at IRF5-TNPO3, 17q12-21 and MMEL1 are associated with primary biliary cirrhosis. Nat Genet 2010;42:655–7. 10.1038/ng.631 20639879 PMC2929126

[48] Liu X, Invernizzi P, Lu Y, et al Genome-wide meta-analyses identify three loci associated with primary biliary cirrhosis. Nat Genet 2010;42:658–60. 10.1038/ng.627 20639880 PMC3150510

[49] Liu JZ, Almarri MA, Gaffney DJ, et al Dense fine-mapping study identifies novel disease loci and implicates coding and non-coding variation in primary biliary cirrhosis risk. Nat Genet 2012;44:1137–41. 22961000 10.1038/ng.2395 PMC3459817

[50] Juran BD, Hirschfield GM, Invernizzi P, et al Immunochip analyses identify a novel risk locus for primary biliary cirrhosis at 13q14, multiple independent associations at four established risk loci and epistasis between 1p31 and 7q32 risk variants. Hum Mol Genet 2012;21:5209–21. 10.1093/hmg/dds359 22936693 PMC3490520

[51] Nakamura M, Nishida N, Kawashima M, et al Genome-wide association study identifies TNFSF15 and POU2AF1 as susceptibility loci for primary biliary cirrhosis in the Japanese population. Am J Hum Genet 2012;91:721–8. 10.1016/j.ajhg.2012.08.010 23000144 PMC3484650

[52] Hirschfield GM, Xie G, Lu E, et al Association of primary biliary cirrhosis with variants in the CLEC16A, SOCS1, SPIB and SIAE immunomodulatory genes. Genes Immun 2012;13:328–35. 10.1038/gene.2011.89 22257840 PMC3360983

[53] Dong M, Li J, Tang R, et al Multiple genetic variants associated with primary biliary cirrhosis in a Han Chinese population. Clin Rev Allergy Immunol 2015;48:316–21. 10.1007/s12016-015-8472-0 25690649 PMC5584624

[54] Cordell HJ, Han Y, Mells GF, et al International genome-wide meta-analysis identifies new primary biliary cirrhosis risk loci and targetable pathogenic pathways. Nat Commun 2015;6:8019 10.1038/ncomms9019 26394269 PMC4580981

[55] Mells GF, Hirschfield GM Making the most of new genetic risk factors: genetic and epigenetic fine mapping of causal autoimmune disease variants. Clin Res Hepatol Gastroenterol 2015;39:408–11. 10.1016/j.clinre.2015.05.002 26160476

[56] Tang R, Chen H, Miao Q, et al The cumulative effects of known susceptibility variants to predict primary biliary cirrhosis risk. Genes Immun 2015;16:238 10.1038/gene.2015.2 25906362

[57] Juran BD, Lazaridis KN Environmental factors in primary biliary cirrhosis. Semin Liver Dis 2014;34:265–72. 10.1055/s-0034-1383726 25057950 PMC4232304

[58] Prince MI, Chetwynd A, Diggle P, et al The geographical distribution of primary biliary cirrhosis in a well-defined cohort. Hepatology 2001;34:1083–8. 10.1053/jhep.2001.29760 11731995

[59] Ala A, Stanca CM, Bu-Ghanim M, et al Increased prevalence of primary biliary cirrhosis near Superfund toxic waste sites. Hepatology 2006;43:525–31. 10.1002/hep.21076 16496326

[60] McNally RJ, Ducker S, James OF Are transient environmental agents involved in the cause of primary biliary cirrhosis? Evidence from space-time clustering analysis. Hepatology 2009;50:1169–74. 10.1002/hep.23139 19711423

[61] Selmi C, Balkwill DL, Invernizzi P, et al Patients with primary biliary cirrhosis react against a ubiquitous xenobiotic-metabolizing bacterium. Hepatology 2003;38:1250–7. 10.1053/jhep.2003.50446 14578864

[62] Burroughs AK, Rosenstein IJ, Epstein O, et al Bacteriuria and primary biliary cirrhosis. Gut 1984;25:133–7. 10.1136/gut.25.2.133 6363217 PMC1432247

[63] Amano K, Leung PS, Rieger R, et al Chemical xenobiotics and mitochondrial autoantigens in primary biliary cirrhosis: identification of antibodies against a common environmental, cosmetic, and food additive, 2-octynoic acid. J Immunol 2005;174:5874–83. 10.4049/jimmunol.174.9.5874 15845458

[64] Sherlock S, Scheuer PJ The presentation and diagnosis of 100 patients with primary biliary cirrhosis. N Engl J Med 1973;289:674–8. 10.1056/NEJM197309272891306 4580473

[65] Prince MI, Chetwynd A, Craig WL, et al Asymptomatic primary biliary cirrhosis: clinical features, prognosis, and symptom progression in a large population based cohort. Gut 2004;53:865–70. 10.1136/gut.2003.023937 15138215 PMC1774078

[66] Marzorati S, Invernizzi P, Lleo A Making sense of autoantibodies in cholestatic liver diseases. Clin Liver Dis 2016;20:33–46. 10.1016/j.cld.2015.08.003 26593289

[67] Walker JG, Doniach D, Roitt IM, et al Serological tests in diagnosis of primary biliary cirrhosis. Lancet 1965;1:827–31. 10.1016/S0140-6736(65)91372-3 14263538

[68] Doniach D, Roitt IM, Walker JG, et al Tissue antibodies in primary biliary cirrhosis, active chronic (lupoid) hepatitis, cryptogenic cirrhosis and other liver diseases and their clinical implications. Clin Exp Immunol 1966;1:237–62. 5330183 PMC1579190

[69] Berg PA, Doniach D, Roitt IM Mitochondrial antibodies in primary biliary cirrhosis. I. Localization of the antigen to mitochondrial membranes. J Exp Med 1967;126:277–90. 10.1084/jem.126.2.277 4165742 PMC2138313

[70] Ruffatti A, Arslan P, Floreani A, et al Nuclear membrane-staining antinuclear antibody in patients with primary biliary cirrhosis. J Clin Immunol 1985;5:357–61. 10.1007/BF00918255 2414313

[71] Lozano F, Parés A, Borche L, et al Autoantibodies against nuclear envelope-associated proteins in primary biliary cirrhosis. Hepatology 1988;8:930–8. 10.1002/hep.1840080438 3292364

[72] Lassoued K, Brenard R, Degos F, et al Antinuclear antibodies directed to a 200-kilodalton polypeptide of the nuclear envelope in primary biliary cirrhosis. A clinical and immunological study of a series of 150 patients with primary biliary cirrhosis. Gastroenterology 1990;99:181–6. 2188869 10.1016/0016-5085(90)91246-3

[73] Nickowitz RE, Wozniak RW, Schaffner F, et al Autoantibodies against integral membrane proteins of the nuclear envelope in patients with primary biliary cirrhosis. Gastroenterology 1994;106:193–9. 10.1016/S0016-5085(94)95333-3 8276182

[74] Fussey SP, Guest JR, James OF, et al Identification and analysis of the major M2 autoantigens in primary biliary cirrhosis. Proc Natl Acad Sci U S A 1988;85:8654–8. 10.1073/pnas.85.22.8654 3186751 PMC282518

[75] Moteki S, Leung PS, Coppel RL, et al Use of a designer triple expression hybrid clone for three different lipoyl domain for the detection of antimitochondrial autoantibodies. Hepatology 1996;24:97–103. 10.1002/hep.510240117 8707289

[76] Nakamura M, Kondo H, Mori T, et al Anti-gp210 and anti-centromere antibodies are different risk factors for the progression of primary biliary cirrhosis. Hepatology 2007;45:118–27. 10.1002/hep.21472 17187436

[77] Nakamura M Clinical significance of autoantibodies in primary biliary cirrhosis. Semin Liver Dis 2014;34:334–40. 10.1055/s-0034-1383732 25057956

[78] Norman GL, Yang CY, Ostendorff HP, et al Anti-kelch-like 12 and anti-hexokinase 1: novel autoantibodies in primary biliary cirrhosis. Liver Int 2015;35:642–51. 10.1111/liv.12690 25243383 PMC4305042

[79] Nakamura M, Kondo H, Tanaka A, et al Autoantibody status and histological variables influence biochemical response to treatment and long-term outcomes in Japanese patients with primary biliary cirrhosis. Hepatol Res 2015;45:846–55. 10.1111/hepr.12423 25220608

[80] Yang WH, Yu JH, Nakajima A, et al Do antinuclear antibodies in primary biliary cirrhosis patients identify increased risk for liver failure? Clin Gastroenterol Hepatol 2004;2:1116–22. 10.1016/S1542-3565(04)00465-3 15625657

[81] Granito A, Muratori P, Muratori L, et al Antibodies to SS-A/Ro-52kD and centromere in autoimmune liver disease: a clue to diagnosis and prognosis of primary biliary cirrhosis. Aliment Pharmacol Ther 2007;26:831–8. 10.1111/j.1365-2036.2007.03433.x 17767467

[82] Rigopoulou EI, Davies ET, Pares A, et al Prevalence and clinical significance of isotype specific antinuclear antibodies in primary biliary cirrhosis. Gut 2005;54:528–32. 10.1136/gut.2003.036558 15753539 PMC1774444

[83] Metcalf JV, Mitchison HC, Palmer JM, et al Natural history of early primary biliary cirrhosis. The Lancet 1996;348:1399–402. 10.1016/S0140-6736(96)04410-8 8937278

[84] Mitchison HC, Bassendine MF, Hendrick A, et al Positive antimitochondrial antibody but normal alkaline phosphatase: is this primary biliary cirrhosis? Hepatology 1986;6:1279–84. 10.1002/hep.1840060609 3793004

[85] Mattalia A, Quaranta S, Leung PS, et al Characterization of antimitochondrial antibodies in healthy adults. Hepatology 1998;27:656–61. 10.1002/hep.510270303 9500690

[86] Kisand KE, Metsküla K, Kisand KV, et al The follow-up of asymptomatic persons with antibodies to pyruvate dehydrogenase in adult population samples. J Gastroenterol 2001;36:248–54. 10.1007/s005350170111 11324728

[87] MacSween RN, Horne CH, Moffat AJ, et al Serum protein levels in primary biliary cirrhosis. J Clin Pathol 1972;25:789–92. 10.1136/jcp.25.9.789 5086222 PMC477512

[88] Taal BG, Schalm SW, de Bruyn AM, et al Serum IgM in primary biliary cirrhosis. Clin Chim Acta 1980;108:457–63. 10.1016/0009-8981(80)90353-8 7471476

[89] Poupon R, Chazouillères O, Balkau B, et al Clinical and biochemical expression of the histopathological lesions of primary biliary cirrhosis. UDCA-PBC Group. J Hepatol 1999;30:408–12. 10.1016/S0168-8278(99)80098-1 10190722

[90] Poupon RE, Balkau B, Eschwège E, et al A multicenter, controlled trial of ursodiol for the treatment of primary biliary cirrhosis. UDCA-PBC Study Group. N Engl J Med 1991;324:1548–54. 10.1056/NEJM199105303242204 1674105

[91] Hirschfield GM, Mason A, Luketic V, et al Efficacy of obeticholic acid in patients with primary biliary cirrhosis and inadequate response to ursodeoxycholic acid. Gastroenterology 2015;148:751–61. 10.1053/j.gastro.2014.12.005 25500425

[92] Rockey DC, Caldwell SH, Goodman ZD, et al American Association for the Study of Liver D. Liver biopsy. Hepatology 2009;49:1017–44. 19243014 10.1002/hep.22742

[93] Nakanuma YZY, Portmann B Diseases of the bile ducts : Burt AD, Ferrell LD, MacSween’s pathology of the liver: Churchill Livingstone, 2012:491–562.

[94] Batts K Autoimmune and chronic cholestatic disorders of the liver : Odze RD, Goldblum JR, Surgical pathology of the GI tract, liver, biliary tract and pancreas. Philadelphia: Elsevier Saunders, 2014.

[95] Degott C, Zafrani ES, Callard P, et al Histopathological study of primary biliary cirrhosis and the effect of ursodeoxycholic acid treatment on histology progression. Hepatology 1999;29:1007–12. 10.1002/hep.510290444 10094939

[96] Corpechot C, Carrat F, Poupon R, et al Primary biliary cirrhosis: incidence and predictive factors of cirrhosis development in ursodiol-treated patients. Gastroenterology 2002;122:652–8. 10.1053/gast.2002.31880 11874998

[97] Garrido MC, Hubscher SG Accuracy of staging in primary biliary cirrhosis. J Clin Pathol 1996;49:556–9. 10.1136/jcp.49.7.556 8813953 PMC500569

[98] Scheuer PJ Ludwig Symposium on biliary disorders-part II. Pathologic features and evolution of primary biliary cirrhosis and primary sclerosing cholangitis. Mayo Clin Proc 1998;73:179–83. 9473003 10.4065/73.2.179

[99] Hubscher SG, Elias E, Buckels JA, et al Primary biliary cirrhosis. Histological evidence of disease recurrence after liver transplantation. J Hepatol 1993;18:173–84. 8409333 10.1016/s0168-8278(05)80244-2

[100] Hiramatsu K, Aoyama H, Zen Y, et al Proposal of a new staging and grading system of the liver for primary biliary cirrhosis. Histopathology 2006;49:466–78. 10.1111/j.1365-2559.2006.02537.x 17064292

[101] Kakuda Y, Harada K, Sawada-Kitamura S, et al Evaluation of a new histologic staging and grading system for primary biliary cirrhosis in comparison with classical systems. Hum Pathol 2013;44:1107–17. 10.1016/j.humpath.2012.09.017 23313306

[102] Chan AW, Chan RC, Wong GL, et al Evaluation of histological staging systems for primary biliary cirrhosis: correlation with clinical and biochemical factors and significance of pathological parameters in prognostication. Histopathology 2014;65:174–86. 10.1111/his.12384 24479738

[103] Wendum D, Boëlle PY, Bedossa P, et al Primary biliary cirrhosis: proposal for a new simple histological scoring system. Liver Int 2015;35:652–9. 10.1111/liv.12620 24939754

[104] Khan FM, Komarla AR, Mendoza PG, et al Keratin 19 demonstration of canal of Hering loss in primary biliary cirrhosis: "minimal change PBC"? Hepatology 2013;57:700–7. 10.1002/hep.26020 22911653

[105] Mayo MJ, Parkes J, Adams-Huet B, et al Prediction of clinical outcomes in primary biliary cirrhosis by serum enhanced liver fibrosis assay. Hepatology 2008;48:1549–57. 10.1002/hep.22517 18846542 PMC2597274

[106] Corpechot C, El Naggar A, Poujol-Robert A, et al Assessment of biliary fibrosis by transient elastography in patients with PBC and PSC. Hepatology 2006;43:1118–24. 10.1002/hep.21151 16628644

[107] Corpechot C, Carrat F, Poujol-Robert A, et al Noninvasive elastography-based assessment of liver fibrosis progression and prognosis in primary biliary cirrhosis. Hepatology 2012;56:198–208. 10.1002/hep.25599 22271046

[108] Carbone M, Sharp SJ, Flack S, et al The UK-PBC risk scores: Derivation and validation of a scoring system for long-term prediction of end-stage liver disease in primary biliary cholangitis. Hepatology 2016;63:930–50. 10.1002/hep.28017 26223498 PMC6984963

[109] Lammers WJ, Hirschfield GM, Corpechot C, et al Development and validation of a scoring system to predict outcomes of patients with primary biliary cirrhosis receiving ursodeoxycholic acid therapy. Gastroenterology 2015;149:1804–12. 10.1053/j.gastro.2015.07.061 26261009

[110] Hirschfield GM Diagnosis of primary biliary cirrhosis. Best Pract Res Clin Gastroenterol 2011;25:701–12. 10.1016/j.bpg.2011.10.005 22117636

[111] Guañabens N, Cerdá D, Monegal A, et al Low bone mass and severity of cholestasis affect fracture risk in patients with primary biliary cirrhosis. Gastroenterology 2010;138:2348–56. 10.1053/j.gastro.2010.02.016 20178794

[112] Guañabens N, Parés A, Ros I, et al Severity of cholestasis and advanced histological stage but not menopausal status are the major risk factors for osteoporosis in primary biliary cirrhosis. J Hepatol 2005;42:573–7. 10.1016/j.jhep.2004.11.035 15763344

[113] Menon KV, Angulo P, Weston S, et al Bone disease in primary biliary cirrhosis: independent indicators and rate of progression. J Hepatol 2001;35:316–23. 10.1016/S0168-8278(01)00144-1 11592591

[114] Varyani FK, West J, Card TR An increased risk of urinary tract infection precedes development of primary biliary cirrhosis. BMC Gastroenterol 2011;11:95 10.1186/1471-230X-11-95 21871059 PMC3175196

[115] Crippin JS, Lindor KD, Jorgensen R, et al Hypercholesterolemia and atherosclerosis in primary biliary cirrhosis: What is the risk? Hepatology 1992;15:858–62. 10.1002/hep.1840150518 1568727

[116] Hiraoka H, Yamashita S, Matsuzawa Y, et al Decrease of hepatic triglyceride lipase levels and increase of cholesteryl ester transfer protein levels in patients with primary biliary cirrhosis: relationship to abnormalities in high-density lipoprotein. Hepatology 1993;18:103–10. 10.1002/hep.1840180117 8325601

[117] Solaymani-Dodaran M, Aithal GP, Card T, et al Risk of cardiovascular and cerebrovascular events in primary biliary cirrhosis: a population-based cohort study. Am J Gastroenterol 2008;103:2784–8. 10.1111/j.1572-0241.2008.02092.x 18759822

[118] Longo M, Crosignani A, Battezzati PM, et al Hyperlipidaemic state and cardiovascular risk in primary biliary cirrhosis. Gut 2002;51:265–9. 10.1136/gut.51.2.265 12117892 PMC1773333

[119] Abu Rajab M, Kaplan MM Statins in primary biliary cirrhosis: are they safe? Dig Dis Sci 2010;55:2086–8. 10.1007/s10620-009-0988-9 19795210

[120] Chalasani N, Bonkovsky HL, Fontana R, et al Features and outcomes of 899 patients with drug-induced liver injury: the DILIN Prospective Study. Gastroenterology 2015;148:1340–52. 10.1053/j.gastro.2015.03.006 25754159 PMC4446235

[121] Stojakovic T, Putz-Bankuti C, Fauler G, et al Atorvastatin in patients with primary biliary cirrhosis and incomplete biochemical response to ursodeoxycholic acid. Hepatology 2007;46:776–84. 10.1002/hep.21741 17668874

[122] Corpechot C, Gaouar F, Chrétien Y, et al Smoking as an independent risk factor of liver fibrosis in primary biliary cirrhosis. J Hepatol 2012;56:218–24. 10.1016/j.jhep.2011.03.031 21703179

[123] Zein CO, Beatty K, Post AB, et al Smoking and increased severity of hepatic fibrosis in primary biliary cirrhosis: a cross validated retrospective assessment. Hepatology 2006;44:1564–71. 10.1002/hep.21423 17133468

[124] Long RG, Scheuer PJ, Sherlock S Presentation and course of asymptomatic primary biliary cirrhosis. Gastroenterology 1977;72:1204–7. 870368

[125] Balasubramaniam K, Grambsch PM, Wiesner RH, et al Diminished survival in asymptomatic primary biliary cirrhosis. A prospective study. Gastroenterology 1990;98:1567–71. 2338193 10.1016/0016-5085(90)91091-j

[126] Springer J, Cauch-Dudek K, O’Rourke K, et al Asymptomatic primary biliary cirrhosis: a study of its natural history and prognosis. Am J Gastroenterol 1999;94:47–53. 10.1111/j.1572-0241.1999.00770.x 9934730

[127] Vleggaar FP, van Buuren HR, Zondervan PE, et al Jaundice in non-cirrhotic primary biliary cirrhosis: the premature ductopenic variant. Gut 2001;49:276–81. 10.1136/gut.49.2.276 11454806 PMC1728410

[128] Bogdanos DP, Liaskos C, Pares A, et al Anti-gp210 antibody mirrors disease severity in primary biliary cirrhosis. Hepatology 2007;45:1583 10.1002/hep.21678 17538935

[129] Rigamonti C, Shand LM, Feudjo M, et al Clinical features and prognosis of primary biliary cirrhosis associated with systemic sclerosis. Gut 2006;55:388–94. 10.1136/gut.2005.075002 16150855 PMC1856066

[130] Dickson ER, Grambsch PM, Fleming TR, et al Prognosis in primary biliary cirrhosis: model for decision making. Hepatology 1989;10:1–7. 10.1002/hep.1840100102 2737595

[131] Chan CW, Carpenter JR, Rigamonti C, et al Survival following the development of ascites and/or peripheral oedema in primary biliary cirrhosis: a staged prognostic model. Scand J Gastroenterol 2005;40:1081–9. 10.1080/00365520510023215 16211715

[132] Shapiro JM, Smith H, Schaffner F Serum bilirubin: a prognostic factor in primary biliary cirrhosis. Gut 1979;20:137–40. 10.1136/gut.20.2.137 428825 PMC1419455

[133] Kilmurry MR, Heathcote EJ, Cauch-Dudek K, et al Is the Mayo model for predicting survival useful after the introduction of ursodeoxycholic acid treatment for primary biliary cirrhosis? Hepatology 1996;23:1148–53. 10.1002/hep.510230532 8621147

[134] Trivedi PJ, Corpechot C, Pares A, et al Risk stratification in autoimmune cholestatic liver diseases: opportunities for clinicians and trialists. Hepatology 2016;63:644–59. 10.1002/hep.28128 26290473 PMC4864755

[135] Kamath PS, Wiesner RH, Malinchoc M, et al A model to predict survival in patients with end-stage liver disease. Hepatology 2001;33:464–70. 10.1053/jhep.2001.22172 11172350

[136] Malinchoc M, Kamath PS, Gordon FD, et al A model to predict poor survival in patients undergoing transjugular intrahepatic portosystemic shunts. Hepatology 2000;31:864–71. 10.1053/he.2000.5852 10733541

[137] Barber K, Madden S, Allen J, et al Elective liver transplant list mortality: development of a United Kingdom end-stage liver disease score. Transplantation 2011;92:469–76. 10.1097/TP.0b013e318225db4d 21775931

[138] Huet PM, Vincent C, Deslaurier J, et al Portal hypertension and primary biliary cirrhosis: effect of long-term ursodeoxycholic acid treatment. Gastroenterology 2008;135:1552–60. 10.1053/j.gastro.2008.07.019 18722374

[139] Trivedi PJ, Bruns T, Cheung A, et al Optimising risk stratification in primary biliary cirrhosis: AST/platelet ratio index predicts outcome independent of ursodeoxycholic acid response. J Hepatol 2014;60:1249–58. 10.1016/j.jhep.2014.01.029 24548531

[140] Hirschfield GM, Heathcote EJ Primary biliary cirrhosis: evidence-based gastroenterology and hepatology. Oxford, UK: Wiley-Blackwell, 2010.

[141] Poupon RE, Poupon R, Balkau B Ursodiol for the long-term treatment of primary biliary cirrhosis. The UDCA-PBC Study Group. N Engl J Med 1994;330:1342–7. 10.1056/NEJM199405123301903 8152446

[142] Lindor KD, Dickson ER, Baldus WP, et al Ursodeoxycholic acid in the treatment of primary biliary cirrhosis. Gastroenterology 1994;106:1284–90. 10.1016/0016-5085(94)90021-3 8174890

[143] Heathcote EJ, Cauch-Dudek K, Walker V, et al The Canadian multicenter double-blind randomized controlled trial of ursodeoxycholic acid in primary biliary cirrhosis. Hepatology 1994;19:1149–56. 10.1002/hep.1840190512 8175136

[144] Turner IB, Myszor M, Mitchison HC, et al A two year controlled trial examining the effectiveness of ursodeoxycholic acid in primary biliary cirrhosis. J Gastroenterol Hepatol 1994;9:162–8. 10.1111/j.1440-1746.1994.tb01237.x 8003650

[145] Poupon RE, Lindor KD, Cauch-Dudek K, et al Combined analysis of randomized controlled trials of ursodeoxycholic acid in primary biliary cirrhosis. Gastroenterology 1997;113:884–90. 10.1016/S0016-5085(97)70183-5 9287980

[146] Combes B, Carithers RL, Maddrey WC, et al A randomized, double-blind, placebo-controlled trial of ursodeoxycholic acid in primary biliary cirrhosis. Hepatology 1995;22:759–66. 7657280

[147] Van Hoogstraten HJ, De Smet MB, Renooij W, et al A randomized trial in primary biliary cirrhosis comparing ursodeoxycholic acid in daily doses of either 10 mg/kg or 20 mg/kg. Dutch Multicentre PBC Study Group. Aliment Pharmacol Ther 1998;12:965–71. 10.1046/j.1365-2036.1998.00395.x 9798800

[148] Corpechot C, Carrat F, Bonnand AM, et al The effect of ursodeoxycholic acid therapy on liver fibrosis progression in primary biliary cirrhosis. Hepatology 2000;32:1196–9. 10.1053/jhep.2000.20240 11093724

[149] Goulis J, Leandro G, Burroughs AK Randomised controlled trials of ursodeoxycholic-acid therapy for primary biliary cirrhosis: a meta-analysis. Lancet 1999;354:1053–60. 10.1016/S0140-6736(98)11293-X 10509495

[150] Shi J, Wu C, Lin Y, et al Long-term effects of mid-dose ursodeoxycholic acid in primary biliary cirrhosis: a meta-analysis of randomized controlled trials. Am J Gastroenterol 2006;101:1529–38. 10.1111/j.1572-0241.2006.00634.x 16863557

[151] Rudic JS, Poropat G, Krstic MN, et al Ursodeoxycholic acid for primary biliary cirrhosis. Cochrane Database Syst Rev 2012;12:CD000551 10.1002/14651858.CD000551.pub3 23235576 PMC7045744

[152] Beuers U Drug insight: mechanisms and sites of action of ursodeoxycholic acid in cholestasis. Nat Clin Pract Gastroenterol Hepatol 2006;3:318–28. 10.1038/ncpgasthep0521 16741551

[153] Gohlke H, Schmitz B, Sommerfeld A, et al α5 β1-integrins are sensors for tauroursodeoxycholic acid in hepatocytes. Hepatology 2013;57:1117–29. 10.1002/hep.25992 22865233

[154] Beuers U, Trauner M, Jansen P, et al New paradigms in the treatment of hepatic cholestasis: from UDCA to FXR, PXR and beyond. J Hepatol 2015;62:S25–S37. 10.1016/j.jhep.2015.02.023 25920087

[155] Kondrackiene J, Beuers U, Kupcinskas L Efficacy and safety of ursodeoxycholic acid versus cholestyramine in intrahepatic cholestasis of pregnancy. Gastroenterology 2005;129:894–901. 10.1053/j.gastro.2005.06.019 16143129

[156] Bacq Y, Sentilhes L, Reyes HB, et al Efficacy of ursodeoxycholic acid in treating intrahepatic cholestasis of pregnancy: a meta-analysis. Gastroenterology 2012;143:1492–501. 10.1053/j.gastro.2012.08.004 22892336

[157] Trivedi PJ, Hirschfield GM, Gershwin ME Obeticholic acid for the treatment of primary biliary cirrhosis. Expert Rev Clin Pharmacol 2016;9:13–26. 10.1586/17512433.2015.1092381 26549695

[158] Nevens F, Andreone P, Mazzella G, et al A placebo-controlled trial of obeticholic acid in primary biliary cholangitis. N Engl J Med 2016;375:631–43. 10.1056/NEJMoa1509840 27532829

[159] Leuschner M, Maier KP, Schlichting J, et al Oral budesonide and ursodeoxycholic acid for treatment of primary biliary cirrhosis: results of a prospective double-blind trial. Gastroenterology 1999;117:918–25. 10.1016/S0016-5085(99)70351-3 10500075

[160] Rautiainen H, Kärkkäinen P, Karvonen AL, et al Budesonide combined with UDCA to improve liver histology in primary biliary cirrhosis: a three-year randomized trial. Hepatology 2005;41:747–52. 10.1002/hep.20646 15754377

[161] Angulo P, Jorgensen RA, Keach JC, et al Oral budesonide in the treatment of patients with primary biliary cirrhosis with a suboptimal response to ursodeoxycholic acid. Hepatology 2000;31:318–23. 10.1002/hep.510310209 10655252

[162] Ghonem NS, Assis DN, Boyer JL Fibrates and cholestasis. Hepatology 2015;62:635–43. 10.1002/hep.27744 25678132 PMC4515188

[163] Kanda T, Yokosuka O, Imazeki F, et al Bezafibrate treatment: a new medical approach for PBC patients? J Gastroenterol 2003;38:573–8. 10.1007/s00535-002-1102-7 12825134

[164] Hosonuma K, Sato K, Yamazaki Y, et al A prospective randomized controlled study of long-term combination therapy using ursodeoxycholic acid and bezafibrate in patients with primary biliary cirrhosis and dyslipidemia. Am J Gastroenterol 2015;110:423–31. 10.1038/ajg.2015.20 25732417

[165] Lens S, Leoz M, Nazal L, et al Bezafibrate normalizes alkaline phosphatase in primary biliary cirrhosis patients with incomplete response to ursodeoxycholic acid. Liver Int 2014;34:197–203. 10.1111/liv.12290 23998489

[166] Levy C, Peter JA, Nelson DR, et al Pilot study: fenofibrate for patients with primary biliary cirrhosis and an incomplete response to ursodeoxycholic acid. Aliment Pharmacol Ther 2011;33:235–42. 10.1111/j.1365-2036.2010.04512.x 21083674

[167] Grigorian AY, Mardini HE, Corpechot C, et al Fenofibrate is effective adjunctive therapy in the treatment of primary biliary cirrhosis: a meta-analysis. Clin Res Hepatol Gastroenterol 2015;39:296–306. 10.1016/j.clinre.2015.02.011 25882906

[168] Cheung AC, Lapointe-Shaw L, Kowgier M, et al Combined ursodeoxycholic acid (UDCA) and fenofibrate in primary biliary cholangitis patients with incomplete UDCA response may improve outcomes. Aliment Pharmacol Ther 2016;43:283–93. 10.1111/apt.13465 26559762

[169] Hegade VS, Khanna A, Walker LJ, et al Long-term fenofibrate treatment in primary biliary cholangitis improves biochemistry but not the UK-PBC risk score. Dig Dis Sci 2016;61:3037–44. 10.1007/s10620-016-4250-y 27435324

[170] Zhang H, Li S, Yang J, et al A meta-analysis of ursodeoxycholic acid therapy versus combination therapy with corticosteroids for PBC-AIH-overlap syndrome: evidence from 97 monotherapy and 117 combinations. Prz Gastroenterol 2015;10:148–55. 10.5114/pg.2015.51187 26516380 PMC4607695

[171] Mells GF, Pells G, Newton JL, et al Impact of primary biliary cirrhosis on perceived quality of life: the UK-PBC national study. Hepatology 2013;58:273–83. 10.1002/hep.26365 23471852

[172] Jacoby A, Rannard A, Buck D, et al Development, validation, and evaluation of the PBC-40, a disease specific health related quality of life measure for primary biliary cirrhosis. Gut 2005;54:1622–9. 10.1136/gut.2005.065862 15961522 PMC1774759

[173] Newton JL, Bhala N, Burt J, et al Characterisation of the associations and impact of symptoms in primary biliary cirrhosis using a disease specific quality of life measure. J Hepatol 2006;44:776–83. 10.1016/j.jhep.2005.12.012 16487619

[174] Beuers U, Kremer AE, Bolier R, et al Pruritus in cholestasis: facts and fiction. Hepatology 2014;60:399–407. 10.1002/hep.26909 24807046

[175] Hegade VS, Mells GF, Lammert C, et al P1152: a comparative study of pruritus in PBC cohorts from UK, USA and Italy. J Hepatol 2015;62:S785 10.1016/S0168-8278(15)31349-0

[176] Summerfield JA, Elias E, Hungerford GD, et al The biliary system in primary biliary cirrhosis. A study by endoscopic retrograde cholangiopancreatography. Gastroenterology 1976;70:240–3. 1248684

[177] Datta DV, Sherlock S Cholestyramine for long term relief of the pruritus complicating intrahepatic cholestasis. Gastroenterology 1966;50:323–32. 5905351

[178] Rust C, Sauter GH, Oswald M, et al Effect of cholestyramine on bile acid pattern and synthesis during administration of ursodeoxycholic acid in man. Eur J Clin Invest 2000;30:135–9. 10.1046/j.1365-2362.2000.00606.x 10651838

[179] Gong Y, Huang Z, Christensen E, et al Ursodeoxycholic acid for patients with primary biliary cirrhosis: an updated systematic review and meta-analysis of randomized clinical trials using Bayesian approach as sensitivity analyses. Am J Gastroenterol 2007;102:1799–807. 10.1111/j.1572-0241.2007.01235.x 17459023

[180] Kuiper EM, van Erpecum KJ, Beuers U, et al The potent bile acid sequestrant colesevelam is not effective in cholestatic pruritus: results of a double-blind, randomized, placebo-controlled trial. Hepatology 2010;52:1334–40. 10.1002/hep.23821 20683930

[181] Kremer AE, van Dijk R, Leckie P, et al Serum autotaxin is increased in pruritus of cholestasis, but not of other origin, and responds to therapeutic interventions. Hepatology 2012;56:1391–400. 10.1002/hep.25748 22473838

[182] Ghent CN, Carruthers SG Treatment of pruritus in primary biliary cirrhosis with rifampin. Results of a double-blind, crossover, randomized trial. Gastroenterology 1988;94:488–93. 3275568 10.1016/0016-5085(88)90442-8

[183] Bachs L, Parés A, Elena M, et al Comparison of rifampicin with phenobarbitone for treatment of pruritus in biliary cirrhosis. Lancet 1989;1:574–6. 10.1016/S0140-6736(89)91608-5 2564110

[184] Podesta AL, Terg P, Villamil R, et al Treatment of pruritus in primary biliary cirrhosis with rifampicin. Results of a double-blind, cross-over, randomized trial. Gastroenterology 1991;94:488–93. 10.1016/0016-5085(88)90442-8 3275568

[185] Bachs L, Parés A, Elena M, et al Effects of long-term rifampicin administration in primary biliary cirrhosis. Gastroenterology 1992;102:2077–80. 10.1016/0016-5085(92)90335-V 1587427

[186] Tandon P, Rowe BH, Vandermeer B, et al The efficacy and safety of bile acid binding agents, opioid antagonists, or rifampin in the treatment of cholestasis-associated pruritus. Am J Gastroenterol 2007;102:1528–36. 10.1111/j.1572-0241.2007.01200.x 17403073

[187] Khurana S, Singh P Rifampin is safe for treatment of pruritus due to chronic cholestasis: a meta-analysis of prospective randomized-controlled trials. Liver Int 2006;26:943–8. 10.1111/j.1478-3231.2006.01326.x 16953834

[188] Prince MI, Burt AD, Jones DE Hepatitis and liver dysfunction with rifampicin therapy for pruritus in primary biliary cirrhosis. Gut 2002;50:436–9. 10.1136/gut.50.3.436 11839728 PMC1773130

[189] Sampaziotis F, Griffiths WJ Severe coagulopathy caused by rifampicin in patients with primary sclerosing cholangitis and refractory pruritus. Br J Clin Pharmacol 2012;73:826–7. 10.1111/j.1365-2125.2011.04158.x 22122359 PMC3403211

[190] Bergasa NV, Talbot TL, Alling DW, et al A controlled trial of naloxone infusions for the pruritus of chronic cholestasis. Gastroenterology 1992;102:544–9. 10.1016/0016-5085(92)90102-5 1732125

[191] Wolfhagen FH, Sternieri E, Hop WC, et al Oral naltrexone treatment for cholestatic pruritus: a double-blind, placebo-controlled study. Gastroenterology 1997;113:1264–9. 10.1053/gast.1997.v113.pm9322521 9322521

[192] Terg R, Coronel E, Sordá J, et al Efficacy and safety of oral naltrexone treatment for pruritus of cholestasis, a crossover, double blind, placebo-controlled study. J Hepatol 2002;37:717–22. 10.1016/S0168-8278(02)00318-5 12445410

[193] Jones EA, Neuberger J, Bergasa NV Opiate antagonist therapy for the pruritus of cholestasis: the avoidance of opioid withdrawal-like reactions. QJM 2002;95:547–52. 10.1093/qjmed/95.8.547 12145394

[194] McRae CA, Prince MI, Hudson M, et al Pain as a complication of use of opiate antagonists for symptom control in cholestasis. Gastroenterology 2003;125:591–6. 10.1016/S0016-5085(03)00879-5 12891561

[195] Jones EA, Dekker LR Florid opioid withdrawal-like reaction precipitated by naltrexone in a patient with chronic cholestasis. Gastroenterology 2000;118:431–2. 10.1016/S0016-5085(00)70225-3 10648471

[196] Mayo MJ, Handem I, Saldana S, et al Sertraline as a first-line treatment for cholestatic pruritus. Hepatology 2007;45:666–74. 10.1002/hep.21553 17326161

[197] Bergasa NV, McGee M, Ginsburg IH, et al Gabapentin in patients with the pruritus of cholestasis: a double-blind, randomized, placebo-controlled trial. Hepatology 2006;44:1317–23. 10.1002/hep.21370 17058231

[198] Kremer AE, Martens JJ, Kulik W, et al Lysophosphatidic acid is a potential mediator of cholestatic pruritus. Gastroenterology 2010;139:1008–18. 10.1053/j.gastro.2010.05.009 20546739

[199] Hofmann AF, Huet PM Nasobiliary drainage for cholestatic pruritus. Hepatology 2006;43:1170–1. 10.1002/hep.21185 16628631

[200] Leckie P, Tritto G, Mookerjee R, et al ‘Out-patient’ albumin dialysis for cholestatic patients with intractable pruritus. Aliment Pharmacol Ther 2012;35:696–704. 10.1111/j.1365-2036.2012.04994.x 22260552

[201] Decock S, Roelandts R, Steenbergen WV, et al Cholestasis-induced pruritus treated with ultraviolet B phototherapy: an observational case series study. J Hepatol 2012;57:637–41. 10.1016/j.jhep.2012.04.023 22613002

[202] Pusl T, Denk GU, Parhofer KG, et al Plasma separation and anion adsorption transiently relieve intractable pruritus in primary biliary cirrhosis. J Hepatol 2006;45:887–91. 10.1016/j.jhep.2006.08.008 17046095

[203] Parés A, Cisneros L, Salmerón JM, et al Extracorporeal albumin dialysis: a procedure for prolonged relief of intractable pruritus in patients with primary biliary cirrhosis. Am J Gastroenterol 2004;99:1105–10. 10.1111/j.1572-0241.2004.30204.x 15180733

[204] Gross CR, Malinchoc M, Kim WR, et al Quality of life before and after liver transplantation for cholestatic liver disease. Hepatology 1999;29:356–64. 10.1002/hep.510290229 9918910

[205] Huet P-M, Deslauriers J, Faucher C, et al Fatigue, mental health and depression in patients with primary biliary cirrhosis (PBC). Hepatology 1996;24:167A.

[206] Cauch-Dudek K, Abbey S, Stewart DE, et al Fatigue in primary biliary cirrhosis. Gut 1998;43:705–10. 10.1136/gut.43.5.705 9824355 PMC1727314

[207] Huet PM, Deslauriers J, Tran A, et al Impact of fatigue on the quality of life of patients with primary biliary cirrhosis. Am J Gastroenterol 2000;95:760–7. 10.1111/j.1572-0241.2000.01857.x 10710071

[208] Goldblatt J, Taylor PJ, Lipman T, et al The true impact of fatigue in primary biliary cirrhosis: a population study. Gastroenterology 2002;122:1235–41. 10.1053/gast.2002.32993 11984509

[209] Newton JL, Hollingsworth KG, Taylor R, et al Cognitive impairment in primary biliary cirrhosis: symptom impact and potential etiology. Hepatology 2008;48:541–9. 10.1002/hep.22371 18563843

[210] Hollingsworth KG, Newton JL, Taylor R, et al Pilot study of peripheral muscle function in primary biliary cirrhosis: potential implications for fatigue pathogenesis. Clin Gastroenterol Hepatol 2008;6:1041–8. 10.1016/j.cgh.2008.04.013 18691944

[211] Carbone M, Bufton S, Monaco A, et al The effect of liver transplantation on fatigue in patients with primary biliary cirrhosis: a prospective study. J Hepatol 2013;59:490–4. 10.1016/j.jhep.2013.04.017 23628322

[212] Jones DE, Sutcliffe K, Pairman J, et al An integrated care pathway improves quality of life in primary biliary cirrhosis. QJM 2008;101:535–43. 10.1093/qjmed/hcn043 18388154

[213] Hollingsworth KG, Newton JL, Robinson L, et al Loss of capacity to recover from acidosis in repeat exercise is strongly associated with fatigue in primary biliary cirrhosis. J Hepatol 2010;53:155–61. 10.1016/j.jhep.2010.02.022 20447719

[214] Mang FW, Michieletti P, O’Rourke K, et al Primary biliary cirrhosis, sicca complex, and dysphagia. Dysphagia 1997;12:167–70. 10.1007/PL00009532 9190103

[215] Tsifetaki N, Kitsos G, Paschides CA, et al Oral pilocarpine for the treatment of ocular symptoms in patients with Sjögren’s syndrome: a randomised 12 week controlled study. Ann Rheum Dis 2003;62:1204–7. 10.1136/ard.2002.003889 14644860 PMC1754388

[216] Ono M, Takamura E, Shinozaki K, et al Therapeutic effect of cevimeline on dry eye in patients with Sjögren’s syndrome: a randomized, double-blind clinical study. Am J Ophthalmol 2004;138:6–17. 10.1016/j.ajo.2004.02.010 15234277

[217] Kruszka P, O’Brian RJ Diagnosis and management of Sjögren syndrome. Am Fam Physician 2009;79:465–70. 19323359

[218] National Institute for Health and Care Excellence (NICE). Raynaud’s phenomenon. NICE clinical knowledge summary, 2014.

[219] Dyson JK, Wilkinson N, Jopson L, et al The inter-relationship of symptom severity and quality of life in 2055 patients with primary biliary cholangitis. Aliment Pharmacol Ther 2016;44:1039–50. 10.1111/apt.13794 27640331 PMC5082554

[220] Corpechot C Utility of noninvasive markers of fibrosis in cholestatic liver diseases. Clin Liver Dis 2016;20:143–58. 10.1016/j.cld.2015.08.013 26593296

[221] Boursier J, Zarski JP, de Ledinghen V, et al Determination of reliability criteria for liver stiffness evaluation by transient elastography. Hepatology 2013;57:1182–91. 10.1002/hep.25993 22899556

[222] European Association for Study of Liver; Asociacion Latinoamericana para el Estudio del Higado. EASL-ALEH Clinical Practice Guidelines: Non-invasive tests for evaluation of liver disease severity and prognosis. J Hepatol 2015;63:237–64. 10.1016/j.jhep.2015.04.006 25911335

[223] Friedrich-Rust M, Müller C, Winckler A, et al Assessment of liver fibrosis and steatosis in PBC with FibroScan, MRI, MR-spectroscopy, and serum markers. J Clin Gastroenterol 2010;44:58–65. 10.1097/MCG.0b013e3181a84b8d 19581812

[224] Bruix J, Sherman M American Association for the Study of Liver D. Management of hepatocellular carcinoma: an update. Hepatology 2011;53:1020–2. 21374666 10.1002/hep.24199 PMC3084991

[225] European Association for the Study of the Liver; European Organisation for Research and Treatment of Cancer. EASL-EORTC clinical practice guidelines: management of hepatocellular carcinoma. J Hepatol 2012;56:908–43. 10.1016/j.jhep.2011.12.001 22424438

[226] Roeb E [Hepatocellular carcinoma - current aspects of screening, surveillance and therapeutic strategies (revised EASL-EORTC recommendations)]. Zentralbl Chir 2014;139:175–83. 10.1055/s-0033-1350669 24132679

[227] Caballería L, Parés A, Castells A, et al Hepatocellular carcinoma in primary biliary cirrhosis: similar incidence to that in hepatitis C virus-related cirrhosis. Am J Gastroenterol 2001;96:1160–3. 10.1111/j.1572-0241.2001.03695.x 11316164

[228] Tomiyama Y, Takenaka K, Kodama T, et al Risk factors for survival and the development of hepatocellular carcinoma in patients with primary biliary cirrhosis. Intern Med 2013;52:1553–9. 10.2169/internalmedicine.52.0010 23857086

[229] Shibuya A, Tanaka K, Miyakawa H, et al Hepatocellular carcinoma and survival in patients with primary biliary cirrhosis. Hepatology 2002;35:1172–8. 10.1053/jhep.2002.33157 11981767

[230] Kuiper EM, Hansen BE, Adang RP, et al Relatively high risk for hepatocellular carcinoma in patients with primary biliary cirrhosis not responding to ursodeoxycholic acid. Eur J Gastroenterol Hepatol 2010;22:1–502. 10.1097/MEG.0b013e32834059e7 21389798

[231] de Franchis R Expanding consensus in portal hypertension: Report of the Baveno VI Consensus Workshop: stratifying risk and individualizing care for portal hypertension. J Hepatol 2015;63:743–52. 26047908 10.1016/j.jhep.2015.05.022

[232] Tripathi D, Stanley AJ, Hayes PC, et al UK guidelines on the management of variceal haemorrhage in cirrhotic patients. Gut 2015;64:1680–704. 10.1136/gutjnl-2015-309262 25887380 PMC4680175

[233] National Institute for Health and Care Excellence (NICE). Cirrhosis in over 16s: assessment and management. London, 2016. 27441331

[234] Navasa M, Parés A, Bruguera M, et al Portal hypertension in primary biliary cirrhosis. Relationship with histological features. J Hepatol 1987;5:292–8. 3429837 10.1016/s0168-8278(87)80035-1

[235] Ali AH, Sinakos E, Silveira MG, et al Varices in early histological stage primary biliary cirrhosis. J Clin Gastroenterol 2011;45:e66–e71. 10.1097/MCG.0b013e3181f18c4e 20856137

[236] Ikeda F, Okamoto R, Baba N, et al Prevalence and associated factors with esophageal varices in early primary biliary cirrhosis. J Gastroenterol Hepatol 2012;27:1320–8. 10.1111/j.1440-1746.2012.07114.x 22414162

[237] García-Pagán JC, Caca K, Bureau C, et al Early use of TIPS in patients with cirrhosis and variceal bleeding. N Engl J Med 2010;362:2370–9. 10.1056/NEJMoa0910102 20573925

[238] de Franchis R, Baveno VIF; Baveno VI Faculty. Expanding consensus in portal hypertension. Report of the Baveno VI Consensus Workshop: Stratifying risk and individualizing care for portal hypertension. J Hepatol 2015;63:743–52. 10.1016/j.jhep.2015.05.022 26047908

[239] European Association for the Study of the Liver. EASL clinical practice guidelines on the management of ascites, spontaneous bacterial peritonitis, and hepatorenal syndrome in cirrhosis. J Hepatol 2010;53:397–417. 10.1016/j.jhep.2010.05.004 20633946

[240] Runyon BA; AASLD. Introduction to the revised American Association for the Study of Liver Diseases Practice Guideline management of adult patients with ascites due to cirrhosis 2012. Hepatology 2013;57:1651–3. 10.1002/hep.26359 23463403

[241] Bureau C, Thabut D, Oberti F, et al Transjugular intrahepatic portosystemic shunts with covered stents increase transplant-free survival of patients with cirrhosis and recurrent ascites. Gastroenterology 2017;152:157–63. 10.1053/j.gastro.2016.09.016 27663604

[242] National Institute for Health and Care Excellence (NICE). Rifaximin for preventing episodes of overt hepatic encephalopathy: Technology appraisal guidance, 2015.

[243] Bass NM, Mullen KD, Sanyal A, et al Rifaximin treatment in hepatic encephalopathy. N Engl J Med 2010;362:1071–81. 10.1056/NEJMoa0907893 20335583

[244] Kimer N, Krag A, Møller S, et al Systematic review with meta-analysis: the effects of rifaximin in hepatic encephalopathy. Aliment Pharmacol Ther 2014;40:123–32. 10.1111/apt.12803 24849268

[245] Bajaj JS Review article: the modern management of hepatic encephalopathy. Aliment Pharmacol Ther 2010;31:537–47. 10.1111/j.1365-2036.2009.04211.x 20002027

[246] Carbone M, Neuberger JM Autoimmune liver disease, autoimmunity and liver transplantation. J Hepatol 2014;60:210–23. 10.1016/j.jhep.2013.09.020 24084655

[247] Neuberger J, Gimson A, Davies M, et al Selection of patients for liver transplantation and allocation of donated livers in the UK. Gut 2008;57:252–7. 10.1136/gut.2007.131730 17895356

[248] Bosch A, Dumortier J, Maucort-Boulch D, et al Preventive administration of UDCA after liver transplantation for primary biliary cirrhosis is associated with a lower risk of disease recurrence. J Hepatol 2015;63:1449–58. 10.1016/j.jhep.2015.07.038 26282232

[249] Montano-Loza AJ, Wasilenko S, Bintner J, et al Cyclosporine A protects against primary biliary cirrhosis recurrence after liver transplantation. Am J Transplant 2010;10:852–8. 10.1111/j.1600-6143.2009.03006.x 20132169

[250] Agmon-Levin N, Kopilov R, Selmi C, et al Vitamin D in primary biliary cirrhosis, a plausible marker of advanced disease. Immunol Res 2015;61:141–6. 10.1007/s12026-014-8594-0 25424577

[251] Phillips JR, Angulo P, Petterson T, et al Fat-soluble vitamin levels in patients with primary biliary cirrhosis. Am J Gastroenterol 2001;96:2745–50. 10.1111/j.1572-0241.2001.04134.x 11569705

[252] Frith J, Kerr S, Robinson L, et al Primary biliary cirrhosis is associated with falls and significant fall related injury. QJM 2010;103:153–61. 10.1093/qjmed/hcp188 20061369

[253] National Institute for Health and Care Excellence (NICE). Oesteoporosis: fragility fracture risk: osteoporosis: assessing the risk of fragility fracture. London, 2012. 32186835

[254] Guañabens N, Parés A, Ros I, et al Alendronate is more effective than etidronate for increasing bone mass in osteopenic patients with primary biliary cirrhosis. Am J Gastroenterol 2003;98:2268–74. 10.1016/S0002-9270(03)00550-1 14572578

[255] Zein CO, Jorgensen RA, Clarke B, et al Alendronate improves bone mineral density in primary biliary cirrhosis: a randomized placebo-controlled trial. Hepatology 2005;42:762–71. 10.1002/hep.20866 16175618

[256] Guañabens N, Monegal A, Cerdá D, et al Randomized trial comparing monthly ibandronate and weekly alendronate for osteoporosis in patients with primary biliary cirrhosis. Hepatology 2013;58:2070–8. 10.1002/hep.26466 23686738

[257] Parés A, Guañabens N Osteoporosis in primary biliary cirrhosis: pathogenesis and treatment. Clin Liver Dis 2008;12:407–24. 10.1016/j.cld.2008.02.005 18456188

[258] Levy C, Harnois DM, Angulo P, et al Raloxifene improves bone mass in osteopenic women with primary biliary cirrhosis: results of a pilot study. Liver Int 2005;25:117–21. 10.1111/j.1478-3231.2005.01026.x 15698408

[259] Boone RH, Cheung AM, Girlan LM, et al Osteoporosis in primary biliary cirrhosis: a randomized trial of the efficacy and feasibility of estrogen/progestin. Dig Dis Sci 2006;51:1103–12. 10.1007/s10620-006-8015-x 16865577

[260] Dyson JK, Hirschfield GM, Adams DH, et al Novel therapeutic targets in primary biliary cirrhosis. Nat Rev Gastroenterol Hepatol 2015;12:147–58. 10.1038/nrgastro.2015.12 25645973

[261] Dyson JK, Webb G, Hirschfield GM, et al Unmet clinical need in autoimmune liver diseases. J Hepatol 2015;62:208–18. 10.1016/j.jhep.2014.09.010 25234946

[262] Jopson L, Newton JL, Palmer J, et al RITPBC: B-cell depleting therapy (rituximab) as a treatment for fatigue in primary biliary cirrhosis: study protocol for a randomised controlled trial. BMJ Open 2015;5:e007985 10.1136/bmjopen-2015-007985 PMC4550715 26297361

[263] Oertelt S, Rieger R, Selmi C, et al A sensitive bead assay for antimitochondrial antibodies: chipping away at AMA-negative primary biliary cirrhosis. Hepatology 2007;45:659–65. 10.1002/hep.21583 17326160

[264] Liu H, Norman GL, Shums Z, et al PBC screen: an IgG/IgA dual isotype ELISA detecting multiple mitochondrial and nuclear autoantibodies specific for primary biliary cirrhosis. J Autoimmun 2010;35:436–42. 10.1016/j.jaut.2010.09.005 20932720

[265] Bizzaro N, Covini G, Rosina F, et al Overcoming a "probable" diagnosis in antimitochondrial antibody negative primary biliary cirrhosis: study of 100 sera and review of the literature. Clin Rev Allergy Immunol 2012;42:288–97. 10.1007/s12016-010-8234-y 21188646

[266] Turchany JM, Uibo R, Kivik T, et al A study of antimitochondrial antibodies in a random population in Estonia. Am J Gastroenterol 1997;92:124–6. 8995951

[267] Metcalf JV, Mitchison HC, Palmer JM, et al Natural history of early primary biliary cirrhosis. Lancet 1996;348:1399–402. 10.1016/S0140-6736(96)04410-8 8937278

[268] Ben-Ari Z, Czaja AJ Autoimmune hepatitis and its variant syndromes. Gut 2001;49:589–94. 10.1136/gut.49.4.589 11559660 PMC1728469

[269] O’Brien C, Joshi S, Feld JJ, et al Long-term follow-up of antimitochondrial antibody-positive autoimmune hepatitis. Hepatology 2008;48:550–6. 10.1002/hep.22380 18666262

[270] Trivedi PJ, Hirschfield GM Review article: overlap syndromes and autoimmune liver disease. Aliment Pharmacol Ther 2012;36:517–33. 10.1111/j.1365-2036.2012.05223.x 22817525

[271] Haldar D, Hirschfield GM Overlap syndrome: a real syndrome? Clin Liver Dis 2014;3:43–7. 10.1002/cld.317 PMC6448697 30992883

[272] Chazouillères O, Wendum D, Serfaty L, et al Primary biliary cirrhosis-autoimmune hepatitis overlap syndrome: clinical features and response to therapy. Hepatology 1998;28:296–301. 10.1002/hep.510280203 9695990

[273] Boberg KM, Chapman RW, Hirschfield GM, et al Overlap syndromes: the International Autoimmune Hepatitis Group (IAIHG) position statement on a controversial issue. J Hepatol 2011;54:374–85. 10.1016/j.jhep.2010.09.002 21067838

[274] Bonder A, Retana A, Winston DM, et al Prevalence of primary biliary cirrhosis-autoimmune hepatitis overlap syndrome. Clin Gastroenterol Hepatol 2011;9:609–12. 10.1016/j.cgh.2011.03.019 21440668

[275] Yokokawa J, Saito H, Kanno Y, et al Overlap of primary biliary cirrhosis and autoimmune hepatitis: characteristics, therapy, and long term outcomes. J Gastroenterol Hepatol 2010;25:376–82. 10.1111/j.1440-1746.2009.06018.x 19817953

[276] Silveira MG, Talwalkar JA, Angulo P, et al Overlap of autoimmune hepatitis and primary biliary cirrhosis: long-term outcomes. Am J Gastroenterol 2007;102:1244–50. 10.1111/j.1572-0241.2007.01136.x 17319931

[277] Trivedi PJ, Kumagi T, Al-Harthy N, et al Good maternal and fetal outcomes for pregnant women with primary biliary cirrhosis. Clin Gastroenterol Hepatol 2014;12:1179–85. 10.1016/j.cgh.2013.11.030 24321209

[278] Geenes V, Chambers J, Khurana R, et al Rifampicin in the treatment of severe intrahepatic cholestasis of pregnancy. Eur J Obstet Gynecol Reprod Biol 2015;189:59–63. 10.1016/j.ejogrb.2015.03.020 25864112

[279] Alallam A, Barth D, Heathcote EJ Role of plasmapheresis in the treatment of severe pruritus in pregnant patients with primary biliary cirrhosis: case reports. Can J Gastroenterol 2008;22:505–7. 10.1155/2008/969826 18478137 PMC2660806

[280] Pearce RM, Jones DE, Newton JL Development of an evidence-based patient information medium: empowering newly diagnosed patients with primary biliary cirrhosis. J Vis Commun Med 2011;34:4–13. 10.3109/17453054.2011.548794 21381930

[281] Blackburn P, Freeston M, Baker CR, et al The role of psychological factors in the fatigue of primary biliary cirrhosis. Liver Int 2007;27:654–61. 10.1111/j.1478-3231.2007.01500.x 17498251

[282] Angulo P, Lindor KD, Therneau TM, et al Utilization of the Mayo risk score in patients with primary biliary cirrhosis receiving ursodeoxycholic acid. Liver 1999;19:115–21. 10.1111/j.1478-3231.1999.tb00020.x 10220741

[283] Patanwala I, McMeekin P, Walters R, et al A validated clinical tool for the prediction of varices in PBC: the Newcastle Varices in PBC Score. J Hepatol 2013;59:327–35. 10.1016/j.jhep.2013.04.010 23608623

[284] Giannini E, Botta F, Borro P, et al Platelet count/spleen diameter ratio: proposal and validation of a non-invasive parameter to predict the presence of oesophageal varices in patients with liver cirrhosis. Gut 2003;52:1200–5. 10.1136/gut.52.8.1200 12865282 PMC1773759

[285] Chawla S, Katz A, Attar BM, et al Platelet count/spleen diameter ratio to predict the presence of esophageal varices in patients with cirrhosis: a systematic review. Eur J Gastroenterol Hepatol 2012;24:1–6. 10.1097/MEG.0b013e3283505015 22410714

[286] Groszmann RJ, Garcia-Tsao G, Bosch J, et al Beta-blockers to prevent gastroesophageal varices in patients with cirrhosis. N Engl J Med 2005;353:2254–61. 10.1056/NEJMoa044456 16306522

[287] Ripoll C, Groszmann R, Garcia-Tsao G, et al Hepatic venous pressure gradient predicts clinical decompensation in patients with compensated cirrhosis. Gastroenterology 2007;133:481–8. 10.1053/j.gastro.2007.05.024 17681169

[288] Vizzutti F, Arena U, Romanelli RG, et al Liver stiffness measurement predicts severe portal hypertension in patients with HCV-related cirrhosis. Hepatology 2007;45:1290–7. 10.1002/hep.21665 17464971

[289] Bureau C, Metivier S, Peron JM, et al Transient elastography accurately predicts presence of significant portal hypertension in patients with chronic liver disease. Aliment Pharmacol Ther 2008;27:1261–8. 10.1111/j.1365-2036.2008.03701.x 18397389

[290] Lemoine M, Katsahian S, Ziol M, et al Liver stiffness measurement as a predictive tool of clinically significant portal hypertension in patients with compensated hepatitis C virus or alcohol-related cirrhosis. Aliment Pharmacol Ther 2008;28:1102–10. 10.1111/j.1365-2036.2008.03825.x 18691352

[291] Frith J, Day CP, Robinson L, et al Potential strategies to improve uptake of exercise interventions in non-alcoholic fatty liver disease. J Hepatol 2010;52:112–6. 10.1016/j.jhep.2009.10.010 19897272

[292] Sokol RJ, Kim YS, Hoofnagle JH, et al Intestinal malabsorption of vitamin E in primary biliary cirrhosis. Gastroenterology 1989;96:479–86. 10.1016/0016-5085(89)91574-6 2910763

[293] Levy C, Lindor KD Management of osteoporosis, fat-soluble vitamin deficiencies, and hyperlipidemia in primary biliary cirrhosis. Clin Liver Dis 2003;7:901–10. 10.1016/S1089-3261(03)00097-7 14594136

[294] Howel D, Fischbacher CM, Bhopal RS, et al An exploratory population-based case-control study of primary biliary cirrhosis. Hepatology 2000;31:1055–60. 10.1053/he.2000.7050 10796879

[295] Floreani A, Spinazzè A, Caballeria L, et al Extrahepatic malignancies in primary biliary cirrhosis: a comparative study at two European centers. Clin Rev Allergy Immunol 2015;48:254–62. 10.1007/s12016-014-8446-7 25205363

[296] Jones DE, Metcalf JV, Collier JD, et al Hepatocellular carcinoma in primary biliary cirrhosis and its impact on outcomes. Hepatology 1997;26:1138–42. 10.1002/hep.510260508 9362353

[297] Trivedi PJ, Lammers WJ, van Buuren HR, et al Stratification of hepatocellular carcinoma risk in primary biliary cirrhosis: a multicentre international study. Gut 2016;65:321–9. 10.1136/gutjnl-2014-308351 25567117

[298] Howel D, Metcalf JV, Gray J, et al Cancer risk in primary biliary cirrhosis: a study in northern England. Gut 1999;45:756–60. 10.1136/gut.45.5.756 10517916 PMC1727737

[299] Crippin JS, Lindor KD, Jorgensen R, et al Hypercholesterolemia and atherosclerosis in primary biliary cirrhosis: what is the risk? Hepatology 1992;15:858–62. 10.1002/hep.1840150518 1568727

[300] Sorokin A, Brown JL, Thompson PD Primary biliary cirrhosis, hyperlipidemia, and atherosclerotic risk: a systematic review. Atherosclerosis 2007;194:293–9. 10.1016/j.atherosclerosis.2006.11.036 17240380

